# Active Interpersonal Touch Gives Rise to the Social Softness Illusion

**DOI:** 10.1016/j.cub.2015.07.049

**Published:** 2015-09-21

**Authors:** Antje Gentsch, Elena Panagiotopoulou, Aikaterini Fotopoulou

**Affiliations:** 1 Research Department of Clinical, Educational and Health Psychology, University College London, London WC1E 6BT, UK

## Abstract

Social touch plays a powerful role in human life, with important physical and mental health benefits in development and adulthood. Touch is central in building the foundations of social interaction, attachment, and cognition [1–5], and early, social touch has unique, beneficial neurophysiological and epigenetic effects [6–9]. The recent discovery of a separate neurophysiological system for affectively laden touch in humans has further kindled scientific interest in the area [10, 11]. Remarkably, however, little is known about what motivates and sustains the human tendency to touch others in a pro-social manner. Given the importance of social touch, we hypothesized that active stroking elicits more sensory pleasure when touching others’ skin than when touching one’s own skin. In a set of six experiments (total N = 133) we found that healthy participants, mostly tested in pairs to account for any objective differences in skin softness, consistently judged another’s skin as feeling softer and smoother than their own skin. We further found that this softness illusion appeared selectively when the touch activated a neurophysiological system for affective touch in the receiver. We conclude that this sensory illusion underlies a novel, bodily mechanism of socio-affective bonding and enhances our motivation to touch others.

## Highlights

• The social softness illusion reflects the sensory hedonics of active social touch
• Stroking others’ skin feels softer and smoother than stroking our own skin
• The illusion appears when the touch activates the receiver’s affective touch system
• The illusion reflects a tactile mechanism of emotional sharing between individuals

## Teaser

Gentsch et al. report a novel illusion—the social softness illusion—revealing that we habitually perceive others’ skin as being softer than our own skin. The illusion reflects a tactile mechanism of emotional sharing between individuals and demonstrates the hedonic benefits of social touch in humans.

## Results and Discussion

Active, interpersonal touch has the unique property of being reciprocal; one cannot touch another person without being touched back. While a plethora of studies have investigated such dual properties in relation to non-affective, discriminatory touch [12], the affective experience of caressing another individual and its psychological effects for the active individual remain unknown. We predicted that active touch would be associated with more positive sensations when directed to the other versus the self, and such effects would be modeled on the expected characteristics of the receiver’s affective experience, i.e., would be modeled upon the properties of the so-called C-tactile (CT)-afferent system of the touch receiver [10]. Unmyelinated CT afferents in hairy skin have been found to be specifically tuned to human caresses, giving rise to pleasant sensations [10, 11] and projecting to the insular cortex [11, 13], a core brain region for sensing the physiological condition of the body and homeostatic regulation [14].

**Figure 1:**
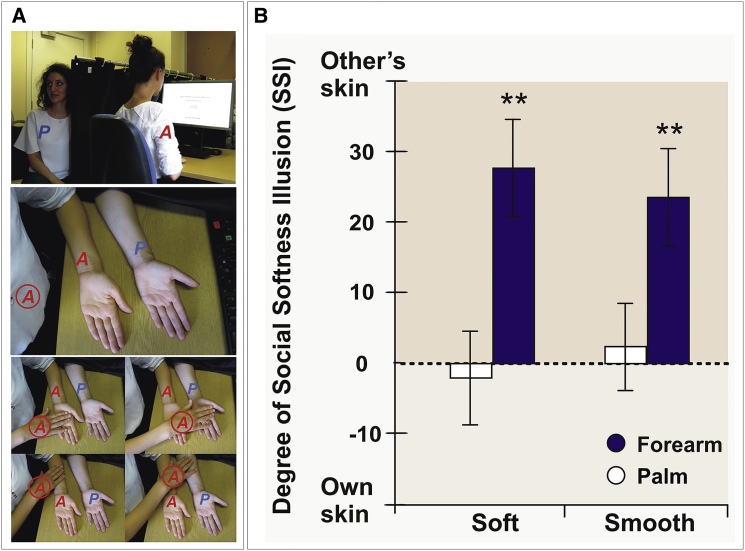
The Social Softness Illusion (A) Experimental setup. The “active” participant (“A”) gently strokes her own left arm (self-touch) versus stroking the left arm of the “passive” participant (“P”; other-touch) with the right hand. (B) Mean ratings of softness and smoothness for one’s own skin relative to another’s skin. When participants were asked to directly compare self-touch and other-touch on a visual analog scale (see also Figure S1), they consistently rated the other’s skin to be softer and smoother than their own skin on the forearm, but not on the palm. This bias is called the social softness illusion (SSI). Error bars represent SEM. ^∗∗^p < 0.01.

In the current study, we tested pairs of healthy participants (N = 133) in a set of six experiments involving independent samples. In all experiments, participants were asked to perform gentle stroking movements on each other’s skin with the index and middle fingers of their right hand (Figure 1A) and to compare directly between the felt softness, smoothness, and comfort of their own skin and of the other person’s skin (see Supplemental Information for the selection of these verbal labels that have been shown to distinctively capture the different composite facets of tactile pleasure) [15, 16]. For each of these labels, ratings were made using a single, computerized visual analog scale (VAS, see Figure S1), anchored at “other skin” and “own skin,” with the mid-point representing no felt difference between the two.

### Do We Perceive Another Person’s Skin as Softer Than Our Own?

In experiment 1, we predicted that active touch would be associated with more positive sensory experiences when directed to the other versus the self, particularly in parts of the skin containing hair follicles, because only hairy skin (e.g., the forearm)—but not glabrous skin (e.g., the palm)—is innervated by CT afferents [10, 11]. Accordingly, we asked participants to stroke each other either on the left palm or the left forearm. Consistent with our assumption, participants felt the other participant’s skin to be softer (*t* = −4.00; *P*_*Bonf*_ = 0.002; Figure 1B; see Supplemental Information for full analysis and overall ANOVA results) and smoother (*t* = −3.41; *P*_*Bonf*_ = 0.008) than their own skin when touching the forearm but not the palm (all *P*_*Bonf*_ > 0.76). Ratings of comfort, instead, showed the opposite tendency, with self-touch felt to be more comfortable than other-touch on the palm (*t* = 4.87; *P*_*Bonf*_ < 0.001) but without significant difference on the forearm (*P*_*Bonf*_ > 0.9). Comfort ratings most likely reflect an effect of social context as induced by the experimental setup, such as when initiating skin contact with an unfamiliar person. One explanation of the observed differences between skin types might be the learned emotional significance of touching hairy skin versus glabrous skin, which might have overruled the uncomfortableness of touching an unfamiliar other person. Being touched on the forearm leads to higher hedonic pleasure than being touched on glabrous skin, which is thought to relate to the CT-afferent system [10, 11]. This suggests that the affective meaning of skin contact changes the perceptual experience in the touch-givers themselves, leading to the illusion that the other’s skin is softer than one’s own. This perceptual illusion may appear despite the normal experience of discomfort involved in social touch of a stranger. In the following, we will refer to this phenomenon as the “social softness illusion” (SSI).

**Figure 2:**
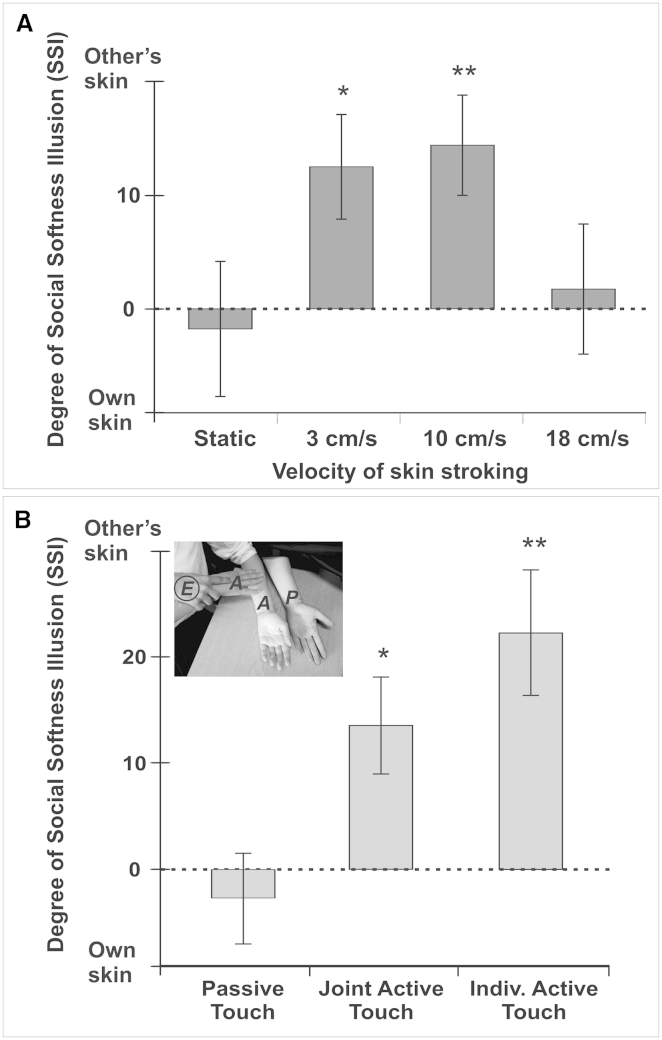
Mediators of the Social Softness Illusion (A) Mean ratings of softness for one’s own skin relative to another’s skin, with the skin stroking being performed at different speeds. The illusory feeling of increased softness of other people’s skin varies in an inverted U-shape pattern with stroking velocity. (B) Experimental setup and mean ratings of softness for one’s own skin relative to another’s skin, with the skin stroking being performed under different conditions of voluntary control. The social softness illusion increased with increasing control over the stroking movement. Together, these results suggest that the social softness illusion is based on affective simulation and sensory prediction based on internal models. Error bars represent SEM. ^∗^p < 0.05, ^∗∗^p < 0.01.

Experiment 2 further investigated the role of stimulating the CT-afferent system in the receiver. We took advantage of the well-established finding that CT afferents are optimally stimulated by light, hairy skin stroking at speeds in the range of 1–10 cm/s, while CT afferent firing reduces at slower or faster tactile stimulation speeds. Therefore, participants were trained to perform either static pressure touch or lateral, dynamic touch movements at 3 cm/s, 10 cm/s, and 18 cm/s speed without vision (Figure S2). The results revealed that the illusion appeared most strongly during slow stroking speeds optimally stimulating CT afferents (at 3 cm/s; *t* = −2.74; *P*_*Bonf*_ = 0.03; 10 cm/s; *t* = −3.69; *P*_*Bonf*_ = 0.004; Figure 2A; see Supplemental Information for full analysis and overall ANOVA results), while it was absent during faster stroking (*P*_*Bonf*_ > 0.9) or during static touch of the skin without relative motion (*P*_*Bonf*_ = 0.77). Thus, the illusion seemed to be dependent on CT-optimal speeds and mirrored the pattern of ratings typically obtained in individuals receiving touch [10]. Additional data from a separate sample of individuals experiencing passive touch confirmed that 3 cm/s and 10 cm/s stroking velocities are rated significantly more pleasant than 18 cm/s touch (*P*_*Bonf*_ < 0.001; see Figure S2). Thus, experiment 2 confirmed that the expectation (even without vision) of inducing a positive bodily state in someone else seems to influence the perception of active touch giving.

### What Are the Sensory and Cognitive Constraints of the Social Softness Illusion?

**Figure 3:**
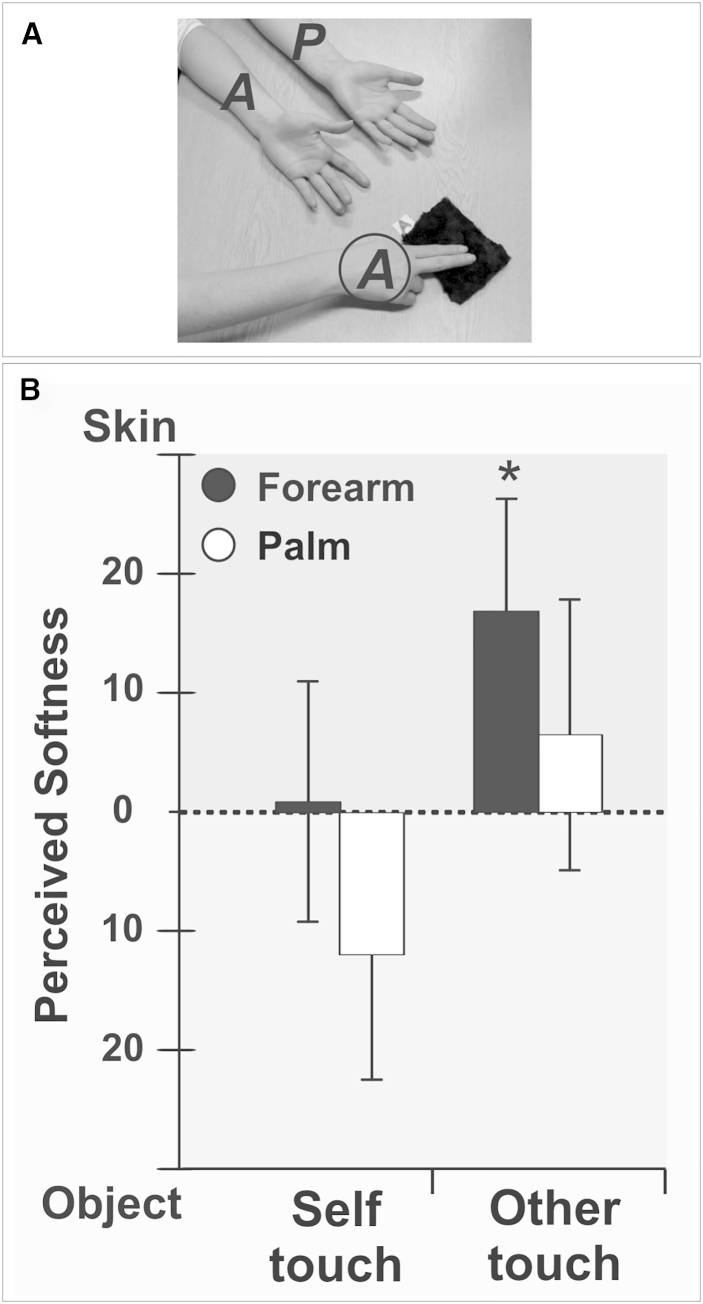
Indirect Assessment of the Social Softness Illusion (A) Experimental setup. Skin softness is indirectly assessed by reference to external objects with surfaces of varying softness. (B) Mean judgments of skin softness relative to surfaces. When asked to compare skin-touch and object-touch, participants rated the other’s skin, but not their own skin, as being softer than the object, but only when touching the forearm and not the palm. Error bars represent SEM. ^∗^p < 0.05.

To exclude the possibility that the SSI was explained by higher-order factors relating to the direct self-other comparison—e.g., social desirability bias, as well as any unaccounted for own-versus-other skin or stroking properties—in experiment 3, we asked a separate sample to compare their own skin and the other’s skin separately, in relation to the texture of external objects. In each trial, they stroked either their own arm or the arm of the other in comparison with one particular object (covered by different textures of increasing softness, see Supplemental Information). The results confirmed that, even relative to external objects of varying softness, participants experienced the other’s skin as being softer than their own skin (*F*(1, 17) = 10.95, p = 0.004; Figure 3). Moreover, this experiment was repeated as a between-subjects design in a new sample of participants. One group of participants compared their own skin to different surfaces, while another group of participants compared another person’s skin to different surfaces, without performing a direct self-other comparison. Under these conditions, the other’s skin was perceived to be smoother than one’s own skin (see Figure S3), ruling out response biases due to social comparison as a possible explanation.

Tactile perception may be influenced by multisensory signals and bottom-up cues regarding the spatial proximity and salience of the touched area. To exclude the possibility that these factors account for the observed SSI in the forearm but not the palm, in experiment 4, we asked a separate sample of paired participants to touch each other’s forearms either at distal sites (lower forearm) or more proximal sites (upper forearm). At both sites, participants reported a significant illusion of experiencing the other’s skin as being softer (proximal site; *t* = −2.91; *P*_*Bonf*_ = 0.02; distal site; *t* = −3.00; *P*_*Bonf*_ = 0.02; Figure S4) and smoother (proximal site; *t* = −3.25; *P*_*Bonf*_ = 0.01; distal site; *t* = −3.09; *P*_*Bonf*_ = 0.02) than their own skin. The tendency for higher comfort experience during self-touch than other-touch was not significant at either site (all *P*_*Bonf*_ > 0.19, see Supplemental Information). These results indicate that spatial proximity and salience cannot explain the SSI.

Finally, experiment 5 tested whether the illusion is specific to skin touch or could be explained by simply approaching and touching the self as compared to the other in these particular body sites. Cotton fabric pieces were attached to the forearms of a new sample of participants, and they were asked to stroke and rate each other’s fabrics. Unbeknownst to them, the fabric textures were identical. Supporting the skin specificity of the SSI, no significant difference in softness or smoothness perception emerged (all *P*_*Bonf*_ > 0.14, see Supplemental Information).

### What Is the Role of Motor Control Mechanisms in the SSI?

Touch is known to be shaped by sensorimotor predictions [17], leading to the reduction of intensity of sensation caused by self-generated movement, a phenomenon well-illustrated by the fact that people cannot tickle themselves [18]. Indeed, self-produced tactile sensations have been shown to be significantly less ticklish, intense, and pleasant than identical stimuli produced by a robot, and progressively so with increasing predictability of the sensory stimulus [17, 19]. Experiment 6 tested the role of these so-called “sensory attenuation” mechanisms [20] in the SSI. Accordingly, we varied how much motor control, and hence sensorimotor predictability, participants had over their stroking action and asked them to compare each other’s skin perception as above. There were two conditions of self-generated touch (active touch conditions), in which participants had full control over the movement, but the touch was performed either individually or jointly with the experimenter. In the joint active condition, the experimenter did not interfere with the participant’s movement but was holding the participant’s arm. This manipulation provided comparable conditions with a passive touch condition in which movements were fully guided and controlled by the experimenter. The social softness illusion appeared during both active touch conditions (individual; *t* = −3.77; *P*_*Bonf*_ = 0.003; joint; *t* = −2.89; *P*_*Bonf*_ = 0.02; Figure 2B; see Supplemental Information for full analysis and overall ANOVA results) but was absent when the skin stroking was passively performed (*P*_*Bonf*_ = 0.54). These results suggest that sensory attenuation mechanisms contribute to the SSI, in the sense that the sensations caused by self-generated movement on another’s skin versus one’s own skin are amplified relative to those caused by passive movement.

However, it should be emphasized that the SSI extends well-established sensory attenuation effects related to self-generated action in at least three important ways. First, to our knowledge this is the first time that sensory attenuation is examined in relation to the perception of sensations generated by actively touching another person versus the self, as opposed to active self-touch being compared to passive touch by another person, or a robot. In the latter—classic sensory attenuation effect—the emphasis is on the relative perceptual attenuation of sensations originating from one’s body, the labeling of actions as self-generated, and related feelings of motor agency [21]. While active, interpersonal touch always entails the aforementioned dual, intertwined experience (you cannot touch another without feeling touch on yourself), the emphasis of the SSI lies in the different sensations derived from actively touching another person versus the self. The experience thus appears centered on the skin of the other person, rather than on one’s own body.

Second, while previous sensory attenuation effects have been observed in both affective (e.g., tickle, tactile pleasure) and emotionally neutral (e.g., tactile discrimination) tactile domains, the SSI is characterized by an affective specificity. Namely, the illusion appears only when one touches another person according to the optimal properties of the latter’s CT-based affective touch system. According to one view, affective touch can be reclassified as interoceptive, similarly to itch and cutaneous pain, given its role in homeostasis and its neurophysiological affinity to other interoceptive pathways [22]. For example, the insular cortex, the main target of the CT projections and known to mediate affective touch signals [11, 13, 23, 24], serves important integrative functions for sensory, motor, and emotional signals and seems to be involved in maintaining the homeostatic control over the body [22, 25, 26].

Third, these two facets of the SSI taken together point to another crucial difference from classic sensory attenuation effects, namely a novel bodily mechanism for social bonding and affiliation. It is well-established that classic sensory attenuation phenomena are affected by top-down factors, such as prior knowledge of agency, assumptions of causation, and interactive action contexts [27–29]. It seems that the SSI is subject to some rather specific socio-affective top-down expectations. Specifically, when active touch is expected to induce a positive, affective state in someone else (CT-specificity of the SSI), the touch-provider experiences an illusory amplification of the sensory pleasure derived from active touch of another individual in relation to the self. These expectations may be informed by prior self, other, or even joint experiences of affective touch pleasure, like those encountered in intimate relations between partners, or between infants and caregivers. They may also be influenced by automatic unconscious simulation of others’ sensations as it occurs during social perception [30]. The posterior insula, for example, has been found to show similar mirror properties during observation of affective touch applied to others [24]. Future studies will need to explore the relation between the SSI phenomenon and emotional perception abilities, perhaps also in different relational contexts. Future studies could also explore the functional connectivity between key structures involved in affective touch, such as the posterior insular cortex [11, 13, 23, 24, 31] and areas that have been shown to modulate the perception of affective touch based on top-down expectations, namely the orbitofrontal cortex [31–34] and the dorsal anterior cingulated cortex [34]. The involvement of the latter brain region, as well as the supplementary motor area [26, 35], may also be linked to the SSI, given their potential role in mediating the relation between action and emotion perception.

Finally, to the best of our knowledge, this novel illusion leads to the first demonstration in humans of the hedonic benefits of social touch for the touch provider. Recent research suggests that softness and smoothness are rewarding tactile attributes that act as reinforcers of behavior and, in opposition to other tactile cues, have been found to produce larger activation in reward and affective brain circuits than in primary sensory areas [32, 36]. This is in line with a long tradition of psychophysical research suggesting soft and smooth textures as pleasant and preferred over rough textures [37–40]. This raises the possibility that the SSI contributes to our motivation to touch each other. While remarkably little is known about what motivates pro-social touch in humans, in other mammals the motivational and functional aspects of a similar, active behavior—namely allogrooming—have been long investigated. Non-human primates are known to spend far more time grooming their conspecifics than they actually need to for hygiene reasons, suggesting that allogrooming and its known beneficial effects on endogenous opioid release [41, 42], pain, and stress alleviation [43] (for review, see [44]), may have a role in promoting social bonds that in turn are important for survival [45]. Future studies could explore similar social bonding effects in humans in relation to the SSI phenomenon.

### Conclusions

In summary, we found that stroking other people’s skin is associated with more positive, sensory experiences than similar self-directed touch, when the stroking is applied voluntarily and according to the optimal properties of the receiver’s CT-based affective touch system. Under these conditions, frequently encountered in intimate relations between partners, or between infants and caregivers, a social softness illusion arises: people have the persistent illusion that others’ skin is softer and smoother than theirs, irrespective of any individual skin differences. Intriguingly, the specificity of the SSI suggests a strong psychological reciprocity in giving and receiving affective touch, providing evidence for a novel, tactile mechanism of emotional sharing between individuals.

## Author Contributions

A.G., E.P., and A.F. conceived the study. A.G. and E.P. collected the data. A.G. conducted the statistical analyses. A.G. and A.F. wrote the paper with input from E.P.

## Supplemental Information

Document S1. Supplemental Experimental Procedures and Figures S1–S3

Document S2. Article plus Supplemental Information
