# Supplementary material for: Active Interpersonal Touch Gives Rise to the Social Softness Illusion
Source: Curr Biol. 2015 Sep 21;25(18):2392–7. doi: 10.1016/j.cub.2015.07.049 (PMC4580302; doi:10.1016/j.cub.2015.07.049)
Supplement: Document S2. Article plus Supplemental Information [file mmc2.pdf]

Report

Current Biology

Active Interpersonal Touch Gives Rise to the Social Softness Illusion

Highlights

- The social softness illusion reflects the sensory hedonics of active social touch
- Stroking others' skin feels softer and smoother than stroking our own skin
- The illusion appears when the touch activates the receiver's affective touch system
- The illusion reflects a tactile mechanism of emotional sharing between individuals

Authors

Antje Gentsch, Elena Panagiotopoulou, Aikaterini Fotopoulou

Correspondence

a.fotopoulou@ucl.ac.uk

In Brief

Gentsch et al. report a novel illusion—the social softness illusion—revealing that we habitually perceive others' skin as being softer than our own skin. The illusion reflects a tactile mechanism of emotional sharing between individuals and demonstrates the hedonic benefits of social touch in humans.

Gentsch et al., 2015, Current Biology *25*, 2392–2397
September 21, 2015 ©2015 The Authors
http://dx.doi.org/10.1016/j.cub.2015.07.049

Current Biology
Report

Active Interpersonal Touch Gives Rise to the Social Softness Illusion

Antje Gentsch,[1] Elena Panagiotopoulou,[1] and Aikaterini Fotopoulou[1,*]
[1]Research Department of Clinical, Educational and Health Psychology, University College London, London WC1E 6BT, UK
*Correspondence: a.fotopoulou@ucl.ac.uk
http://dx.doi.org/10.1016/j.cub.2015.07.049
This is an open access article under the CC BY license (http://creativecommons.org/licenses/by/4.0/).

SUMMARY

Social touch plays a powerful role in human life, with important physical and mental health benefits in development and adulthood. Touch is central in building the foundations of social interaction, attachment, and cognition [1–5], and early, social touch has unique, beneficial neurophysiological and epigenetic effects [6–9]. The recent discovery of a separate neurophysiological system for affectively laden touch in humans has further kindled scientific interest in the area [10, 11]. Remarkably, however, little is known about what motivates and sustains the human tendency to touch others in a pro-social manner. Given the importance of social touch, we hypothesized that active stroking elicits more sensory pleasure when touching others' skin than when touching one's own skin. In a set of six experiments (total N = 133) we found that healthy participants, mostly tested in pairs to account for any objective differences in skin softness, consistently judged another's skin as feeling softer and smoother than their own skin. We further found that this softness illusion appeared selectively when the touch activated a neurophysiological system for affective touch in the receiver. We conclude that this sensory illusion underlies a novel, bodily mechanism of socio-affective bonding and enhances our motivation to touch others.

RESULTS AND DISCUSSION

Active, interpersonal touch has the unique property of being reciprocal; one cannot touch another person without being touched back. While a plethora of studies have investigated such dual properties in relation to non-affective, discriminatory touch [12], the affective experience of caressing another individual and its psychological effects for the active individual remain unknown. We predicted that active touch would be associated with more positive sensations when directed to the other versus the self, and such effects would be modeled on the expected characteristics of the receiver's affective experience, i.e., would be modeled upon the properties of the so-called C-tactile (CT)-afferent system of the touch receiver [10]. Unmyelinated CT afferents in hairy skin have been found to be specifically tuned to human caresses, giving rise to pleasant sensations [10, 11] and projecting to the insular cortex [11, 13], a core brain region for sensing the physiological condition of the body and homeostatic regulation [14].

In the current study, we tested pairs of healthy participants (N = 133) in a set of six experiments involving independent samples. In all experiments, participants were asked to perform gentle stroking movements on each other's skin with the index and middle fingers of their right hand (Figure 1A) and to compare directly between the felt softness, smoothness, and comfort of their own skin and of the other person's skin (see Supplemental Information for the selection of these verbal labels that have been shown to distinctively capture the different composite facets of tactile pleasure) [15, 16]. For each of these labels, ratings were made using a single, computerized visual analog scale (VAS, see Figure S1), anchored at "other skin" and "own skin," with the mid-point representing no felt difference between the two.

Do We Perceive Another Person's Skin as Softer Than Our Own?

In experiment 1, we predicted that active touch would be associated with more positive sensory experiences when directed to the other versus the self, particularly in parts of the skin containing hair follicles, because only hairy skin (e.g., the forearm)—but not glabrous skin (e.g., the palm)—is innervated by CT afferents [10, 11]. Accordingly, we asked participants to stroke each other either on the left palm or the left forearm. Consistent with our assumption, participants felt the other participant's skin to be softer ($t = -4.00$; $P_{Bonf} = 0.002$; Figure 1B; see Supplemental Information for full analysis and overall ANOVA results) and smoother ($t = -3.41$; $P_{Bonf} = 0.008$) than their own skin when touching the forearm but not the palm (all $P_{Bonf} > 0.76$). Ratings of comfort, instead, showed the opposite tendency, with self-touch felt to be more comfortable than other-touch on the palm ($t = 4.87$; $P_{Bonf} < 0.001$) but without significant difference on the forearm ($P_{Bonf} > 0.9$). Comfort ratings most likely reflect an effect of social context as induced by the experimental setup, such as when initiating skin contact with an unfamiliar person. One explanation of the observed differences between skin types might be the learned emotional significance of touching hairy skin versus glabrous skin, which might have overruled the uncomfortableness of touching an unfamiliar other person. Being touched on the forearm leads to higher hedonic pleasure than being touched on glabrous skin, which is thought to relate to the CT-afferent system [10, 11]. This suggests that the affective meaning of skin contact changes the perceptual experience

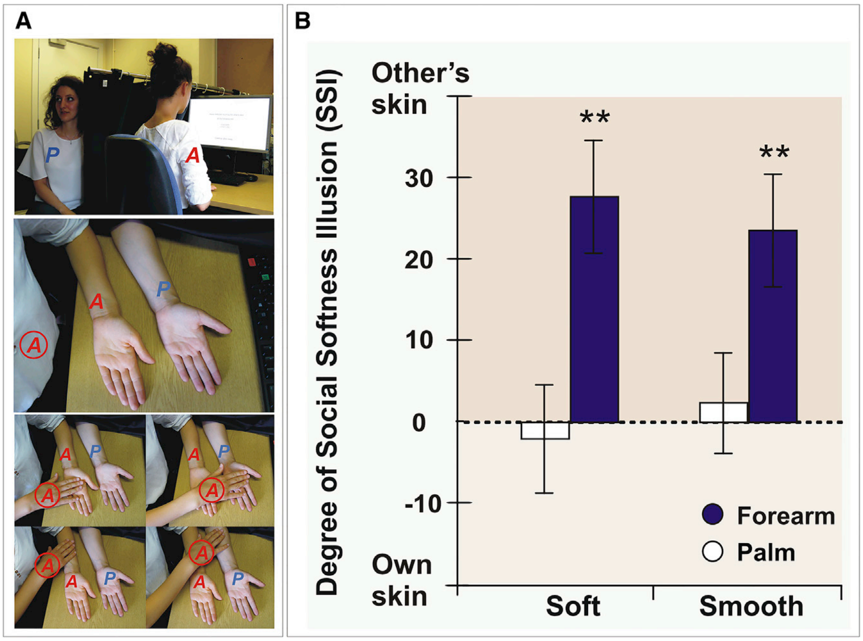

Figure 1. The Social Softness Illusion

(A) Experimental setup. The "active" participant ("A") gently strokes her own left arm (self-touch) versus stroking the left arm of the "passive" participant ("P"; other-touch) with the right hand.

(B) Mean ratings of softness and smoothness for one's own skin relative to another's skin. When participants were asked to directly compare self-touch and other-touch on a visual analog scale (see also Figure S1), they consistently rated the other's skin to be softer and smoother than their own skin on the forearm, but not on the palm. This bias is called the social softness illusion (SSI). Error bars represent SEM. **p < 0.01.

in the touch-givers themselves, leading to the illusion that the other's skin is softer than one's own. This perceptual illusion may appear despite the normal experience of discomfort involved in social touch of a stranger. In the following, we will refer to this phenomenon as the "social softness illusion" (SSI).

Experiment 2 further investigated the role of stimulating the CT-afferent system in the receiver. We took advantage of the well-established finding that CT afferents are optimally stimulated by light, hairy skin stroking at speeds in the range of 1–10 cm/s, while CT afferent firing reduces at slower or faster tactile stimulation speeds. Therefore, participants were trained to perform either static pressure touch or lateral, dynamic touch movements at 3 cm/s, 10 cm/s, and 18 cm/s speed without vision (Figure S2). The results revealed that the illusion appeared most strongly during slow stroking speeds optimally stimulating CT afferents (at 3 cm/s; $t = -2.74$; $P_{Bonf} = 0.03$; 10 cm/s; $t = -3.69$; $P_{Bonf} = 0.004$; Figure 2A; see Supplemental Information for full analysis and overall ANOVA results), while it was absent during faster stroking ($P_{Bonf} > 0.9$) or during static touch of the skin without relative motion ($P_{Bonf} = 0.77$). Thus, the illusion seemed to be dependent on CT-optimal speeds and mirrored the pattern of ratings typically obtained in individuals receiving touch [10]. Additional data from a separate sample of individuals experiencing passive touch confirmed that 3 cm/s and 10 cm/s stroking velocities are rated significantly more pleasant than 18 cm/s touch ($P_{Bonf} < 0.001$; see Figure S2). Thus, experiment 2 confirmed that the expectation (even without vision) of inducing a positive bodily state in someone else seems to influence the perception of active touch giving.

What Are the Sensory and Cognitive Constraints of the Social Softness Illusion?

To exclude the possibility that the SSI was explained by higher-order factors relating to the direct self-other comparison—e.g., social desirability bias, as well as any unaccounted for own-versus-other skin or stroking properties—in experiment 3, we asked a separate sample to compare their own skin and the other's skin separately, in relation to the texture of external objects. In each trial, they stroked either their own arm or the arm of the other in comparison with one particular object (covered by different textures of increasing softness, see Supplemental Information). The results confirmed that, even relative to external objects of varying softness, participants experienced the other's skin as being softer than their own skin ($F(1, 17) = 10.95$, $p = 0.004$; Figure 3). Moreover, this experiment was repeated as a between-subjects design in a new sample of participants. One group of participants compared their own skin to different surfaces, while another group of participants compared another person's skin to different surfaces, without performing a direct self-other comparison. Under these conditions, the other's skin was perceived to be smoother than one's own skin (see Figure S3), ruling out response biases due to social comparison as a possible explanation.

Tactile perception may be influenced by multisensory signals and bottom-up cues regarding the spatial proximity and salience of the touched area. To exclude the possibility that these factors account for the observed SSI in the forearm but not the palm, in experiment 4, we asked a separate sample of paired participants to touch each other's forearms either at distal sites (lower forearm) or more proximal sites (upper forearm). At both sites, participants reported a significant illusion of experiencing the other's skin as being softer (proximal site; $t = -2.91$; $P_{Bonf} = 0.02$; distal site; $t = -3.00$; $P_{Bonf} = 0.02$; Figure S4) and smoother (proximal site; $t = -3.25$; $P_{Bonf} = 0.01$; distal site; $t = -3.09$; $P_{Bonf} = 0.02$) than their own skin. The tendency for higher comfort experience during self-touch than other-touch was not significant at either site (all $P_{Bonf} > 0.19$, see Supplemental Information). These results indicate that spatial proximity and salience cannot explain the SSI.

Finally, experiment 5 tested whether the illusion is specific to skin touch or could be explained by simply approaching and touching the self as compared to the other in these particular body sites. Cotton fabric pieces were attached to the forearms of a new sample of participants, and they were asked to stroke and rate each other's fabrics. Unbeknownst to them, the fabric textures were identical. Supporting the skin specificity of the

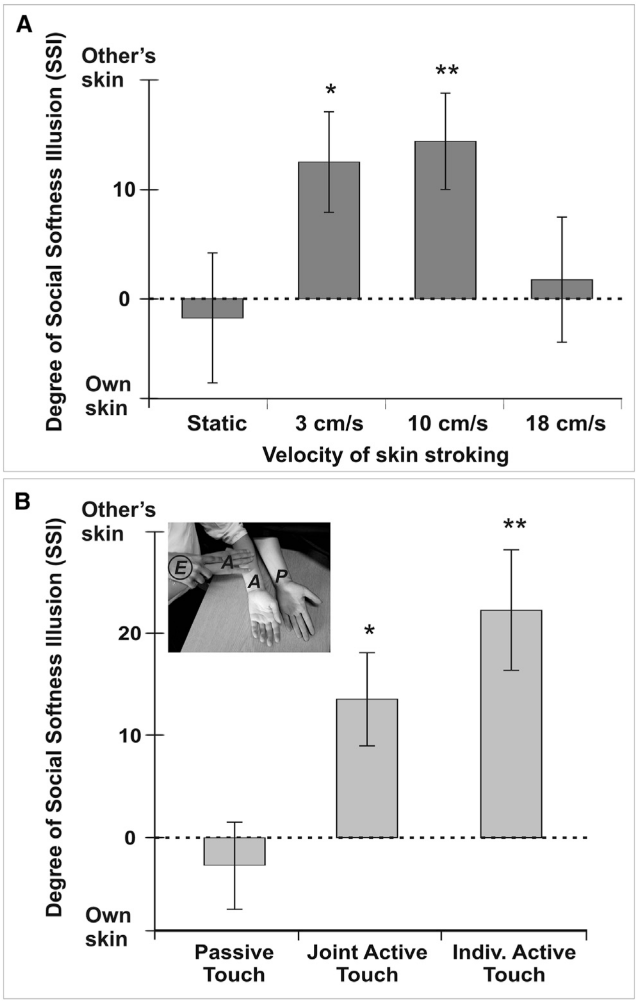

Figure 2. Mediators of the Social Softness Illusion

(A) Mean ratings of softness for one's own skin relative to another's skin, with the skin stroking being performed at different speeds. The illusory feeling of increased softness of other people's skin varies in an inverted U-shape pattern with stroking velocity.

(B) Experimental setup and mean ratings of softness for one's own skin relative to another's skin, with the skin stroking being performed under different conditions of voluntary control. The social softness illusion increased with increasing control over the stroking movement. Together, these results suggest that the social softness illusion is based on affective simulation and sensory prediction based on internal models. Error bars represent SEM. $^{*}p < 0.05$, $^{**}p < 0.01$.

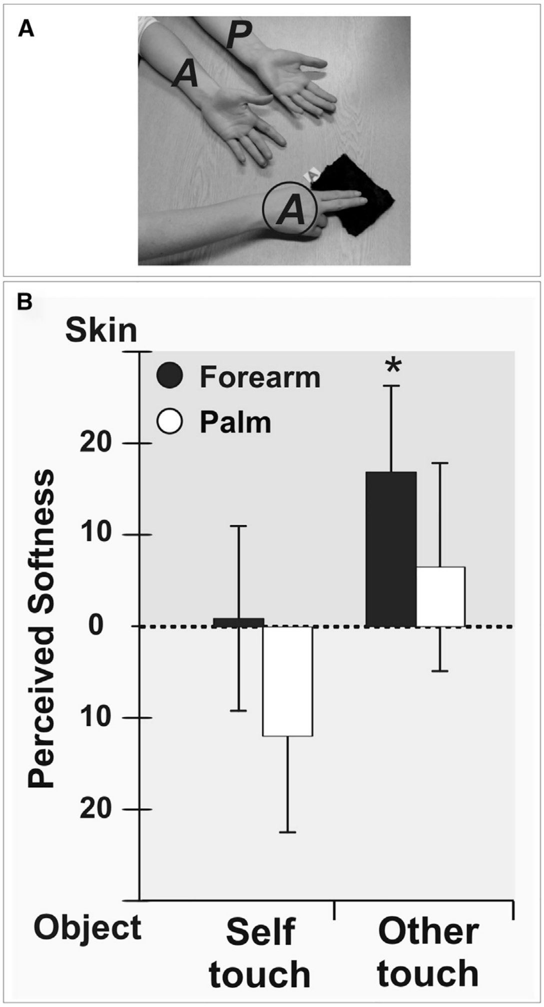

Figure 3. Indirect Assessment of the Social Softness Illusion

(A) Experimental setup. Skin softness is indirectly assessed by reference to external objects with surfaces of varying softness.

(B) Mean judgments of skin softness relative to surfaces. When asked to compare skin-touch and object-touch, participants rated the other's skin, but not their own skin, as being softer than the object, but only when touching the forearm and not the palm. Error bars represent SEM. $^{*}p < 0.05$.

SSI, no significant difference in softness or smoothness perception emerged (all $P_{Bonf} > 0.14$, see Supplemental Information).

What Is the Role of Motor Control Mechanisms in the SSI?

Touch is known to be shaped by sensorimotor predictions [17], leading to the reduction of intensity of sensation caused by self-generated movement, a phenomenon well-illustrated by the fact that people cannot tickle themselves [18]. Indeed, self-produced tactile sensations have been shown to be significantly less ticklish, intense, and pleasant than identical stimuli produced by a robot, and progressively so with increasing predictability of the sensory stimulus [17, 19]. Experiment 6 tested the role of these so-called "sensory attenuation" mechanisms [20] in the SSI. Accordingly, we varied how much motor control, and hence sensorimotor predictability, participants had over their stroking action and asked them to compare each other's skin perception as above. There were two conditions of self-generated touch (active touch conditions), in which participants had full control over the movement, but the touch was performed either individually or jointly with the experimenter. In the joint active condition, the experimenter did not interfere with the participant's movement but was holding the participant's arm. This manipulation provided comparable conditions with a passive touch condition in which movements were fully guided and controlled by the experimenter. The social softness illusion appeared during both active touch conditions (individual;

$t = -3.77$; $P_{Bonf} = 0.003$; joint; $t = -2.89$; $P_{Bonf} = 0.02$; Figure 2B; see Supplemental Information for full analysis and overall ANOVA results) but was absent when the skin stroking was passively performed ($P_{Bonf} = 0.54$). These results suggest that sensory attenuation mechanisms contribute to the SSI, in the sense that the sensations caused by self-generated movement on another's skin versus one's own skin are amplified relative to those caused by passive movement.

However, it should be emphasized that the SSI extends well-established sensory attenuation effects related to self-generated action in at least three important ways. First, to our knowledge this is the first time that sensory attenuation is examined in relation to the perception of sensations generated by actively touching another person versus the self, as opposed to active self-touch being compared to passive touch by another person, or a robot. In the latter—classic sensory attenuation effect—the emphasis is on the relative perceptual attenuation of sensations originating from one's body, the labeling of actions as self-generated, and related feelings of motor agency [21]. While active, interpersonal touch always entails the aforementioned dual, intertwined experience (you cannot touch another without feeling touch on yourself), the emphasis of the SSI lies in the different sensations derived from actively touching another person versus the self. The experience thus appears centered on the skin of the other person, rather than on one's own body.

Second, while previous sensory attenuation effects have been observed in both affective (e.g., tickle, tactile pleasure) and emotionally neutral (e.g., tactile discrimination) tactile domains, the SSI is characterized by an affective specificity. Namely, the illusion appears only when one touches another person according to the optimal properties of the latter's CT-based affective touch system. According to one view, affective touch can be reclassified as interoceptive, similarly to itch and cutaneous pain, given its role in homeostasis and its neurophysiological affinity to other interoceptive pathways [22]. For example, the insular cortex, the main target of the CT projections and known to mediate affective touch signals [11, 13, 23, 24], serves important integrative functions for sensory, motor, and emotional signals and seems to be involved in maintaining the homeostatic control over the body [22, 25, 26].

Third, these two facets of the SSI taken together point to another crucial difference from classic sensory attenuation effects, namely a novel bodily mechanism for social bonding and affiliation. It is well-established that classic sensory attenuation phenomena are affected by top-down factors, such as prior knowledge of agency, assumptions of causation, and interactive action contexts [27–29]. It seems that the SSI is subject to some rather specific socio-affective top-down expectations. Specifically, when active touch is expected to induce a positive, affective state in someone else (CT-specificity of the SSI), the touch-provider experiences an illusory amplification of the sensory pleasure derived from active touch of another individual in relation to the self. These expectations may be informed by prior self, other, or even joint experiences of affective touch pleasure, like those encountered in intimate relations between partners, or between infants and caregivers. They may also be influenced by automatic unconscious simulation of others' sensations as it occurs during social perception [30]. The posterior insula, for example, has been found to show similar mirror properties during observation of affective touch applied to others [24]. Future studies will need to explore the relation between the SSI phenomenon and emotional perception abilities, perhaps also in different relational contexts. Future studies could also explore the functional connectivity between key structures involved in affective touch, such as the posterior insular cortex [11, 13, 23, 24, 31] and areas that have been shown to modulate the perception of affective touch based on top-down expectations, namely the orbitofrontal cortex [31–34] and the dorsal anterior cingulated cortex [34]. The involvement of the latter brain region, as well as the supplementary motor area [26, 35], may also be linked to the SSI, given their potential role in mediating the relation between action and emotion perception.

Finally, to the best of our knowledge, this novel illusion leads to the first demonstration in humans of the hedonic benefits of social touch for the touch provider. Recent research suggests that softness and smoothness are rewarding tactile attributes that act as reinforcers of behavior and, in opposition to other tactile cues, have been found to produce larger activation in reward and affective brain circuits than in primary sensory areas [32, 36]. This is in line with a long tradition of psychophysical research suggesting soft and smooth textures as pleasant and preferred over rough textures [37–40]. This raises the possibility that the SSI contributes to our motivation to touch each other. While remarkably little is known about what motivates pro-social touch in humans, in other mammals the motivational and functional aspects of a similar, active behavior—namely allogrooming—have been long investigated. Non-human primates are known to spend far more time grooming their conspecifics than they actually need to for hygiene reasons, suggesting that allogrooming and its known beneficial effects on endogenous opioid release [41, 42], pain, and stress alleviation [43] (for review, see [44]), may have a role in promoting social bonds that in turn are important for survival [45]. Future studies could explore similar social bonding effects in humans in relation to the SSI phenomenon.

Conclusions

In summary, we found that stroking other people's skin is associated with more positive, sensory experiences than similar self-directed touch, when the stroking is applied voluntarily and according to the optimal properties of the receiver's CT-based affective touch system. Under these conditions, frequently encountered in intimate relations between partners, or between infants and caregivers, a social softness illusion arises: people have the persistent illusion that others' skin is softer and smoother than theirs, irrespective of any individual skin differences. Intriguingly, the specificity of the SSI suggests a strong psychological reciprocity in giving and receiving affective touch, providing evidence for a novel, tactile mechanism of emotional sharing between individuals.

SUPPLEMENTAL INFORMATION

Supplemental Information includes Supplemental Experimental Procedures and three figures and can be found with this article online at http://dx.doi.org/10.1016/j.cub.2015.07.049.

AUTHOR CONTRIBUTIONS

A.G., E.P., and A.F. conceived the study. A.G. and E.P. collected the data. A.G. conducted the statistical analyses. A.G. and A.F. wrote the paper with input from E.P.

ACKNOWLEDGMENTS

The study was approved by the local ethics committee, and participants gave written informed consent. We thank Laura Crucianelli and Yvonne Blake for assistance with data collection, and we would also like to thank the anonymous reviewers for their thoughtful comments. This work was supported by the European Research Council (ERC) Starting Grant ERC-2012-STG GA313755 (to A.F).

Received: May 1, 2015
Revised: June 15, 2015
Accepted: July 20, 2015
Published: September 10, 2015

REFERENCES

1. Field, T. (2003). Touch (Cambridge, MA: MIT Press).
2. Montagu, A. (1971). Touching: The Human Significance of the Skin (New York: Columbia University Press).
3. Harlow, H.F. (1958). The nature of love. Am. Psychol. *13*, 673–685.
4. Gallace, A., and Spence, C. (2010). The science of interpersonal touch: an overview. Neurosci. Biobehav. Rev. *34*, 246–259.
5. Cascio, C.J. (2010). Somatosensory processing in neurodevelopmental disorders. J. Neurodev. Disord. *2*, 62–69.
6. Sharp, H., Pickles, A., Meaney, M., Marshall, K., Tibu, F., and Hill, J. (2012). Frequency of infant stroking reported by mothers moderates the effect of prenatal depression on infant behavioural and physiological outcomes. PLoS ONE *7*, e45446.
7. Bystrova, K., Ivanova, V., Edhborg, M., Matthiesen, A.S., Ransjö-Arvidson, A.B., Mukhamedrakhimov, R., Uvnäs-Moberg, K., and Widström, A.M. (2009). Early contact versus separation: effects on mother-infant interaction one year later. Birth *36*, 97–109.
8. Diamond, A., and Amso, D. (2008). Contributions of Neuroscience to Our Understanding of Cognitive Development. Curr. Dir. Psychol. Sci. *17*, 136–141.
9. Korosi, A., Shanabrough, M., McClelland, S., Liu, Z.W., Borok, E., Gao, X.B., Horvath, T.L., and Baram, T.Z. (2010). Early-life experience reduces excitation to stress-responsive hypothalamic neurons and reprograms the expression of corticotropin-releasing hormone. J. Neurosci. *30*, 703–713.
10. Löken, L.S., Wessberg, J., Morrison, I., McGlone, F., and Olausson, H. (2009). Coding of pleasant touch by unmyelinated afferents in humans. Nat. Neurosci. *12*, 547–548.
11. Olausson, H., Lamarre, Y., Backlund, H., Morin, C., Wallin, B.G., Starck, G., Ekholm, S., Strigo, I., Worsley, K., Vallbo, A.B., and Bushnell, M.C. (2002). Unmyelinated tactile afferents signal touch and project to insular cortex. Nat. Neurosci. *5*, 900–904.
12. Serino, A., and Haggard, P. (2010). Touch and the body. Neurosci. Biobehav. Rev. *34*, 224–236.
13. Olausson, H.W., Cole, J., Vallbo, A., McGlone, F., Elam, M., Krämer, H.H., Rylander, K., Wessberg, J., and Bushnell, M.C. (2008). Unmyelinated tactile afferents have opposite effects on insular and somatosensory cortical processing. Neurosci. Lett. *436*, 128–132.
14. Craig, A.D. (2003). Interoception: the sense of the physiological condition of the body. Curr. Opin. Neurobiol. *13*, 500–505.
15. Guest, S., Dessirier, J.M., Mehrabyan, A., McGlone, F., Essick, G., Gescheider, G., Fontana, A., Xiong, R., Ackerley, R., and Blot, K. (2011). The development and validation of sensory and emotional scales of touch perception. Atten. Percept. Psychophys. *73*, 531–550.
16. Ackerley, R., Saar, K., McGlone, F., and Backlund Wasling, H. (2014). Quantifying the sensory and emotional perception of touch: differences between glabrous and hairy skin. Front. Behav. Neurosci. *8*, 34.
17. Blakemore, S.J., Wolpert, D.M., and Frith, C.D. (1998). Central cancellation of self-produced tickle sensation. Nat. Neurosci. *1*, 635–640.
18. Weiskrantz, L., Elliott, J., and Darlington, C. (1971). Preliminary observations on tickling oneself. Nature *230*, 598–599.
19. Blakemore, S.J., Frith, C.D., and Wolpert, D.M. (1999). Spatio-temporal prediction modulates the perception of self-produced stimuli. J. Cogn. Neurosci. *11*, 551–559.
20. Wolpert, D.M. (1997). Computational approaches to motor control. Trends Cogn. Sci. *1*, 209–216.
21. Blakemore, S.J., Wolpert, D.M., and Frith, C.D. (2002). Abnormalities in the awareness of action. Trends Cogn. Sci. *6*, 237–242.
22. Craig, A.D. (2009). How do you feel–now? The anterior insula and human awareness. Nat. Rev. Neurosci. *10*, 59–70.
23. Björnsdotter, M., Löken, L., Olausson, H., Vallbo, A., and Wessberg, J. (2009). Somatotopic organization of gentle touch processing in the posterior insular cortex. J. Neurosci. *29*, 9314–9320.
24. Morrison, I., Björnsdotter, M., and Olausson, H. (2011). Vicarious responses to social touch in posterior insular cortex are tuned to pleasant caressing speeds. J. Neurosci. *31*, 9554–9562.
25. Paulus, M.P. (2007). Neural basis of reward and craving–a homeostatic point of view. Dialogues Clin. Neurosci. *9*, 379–387.
26. Craig, A.D. (2002). How do you feel? Interoception: the sense of the physiological condition of the body. Nat. Rev. Neurosci. *3*, 655–666.
27. Gentsch, A., and Schütz-Bosbach, S. (2011). I did it: unconscious expectation of sensory consequences modulates the experience of self-agency and its functional signature. J. Cogn. Neurosci. *23*, 3817–3828.
28. Desantis, A., Weiss, C., Schütz-Bosbach, S., and Waszak, F. (2012). Believing and perceiving: authorship belief modulates sensory attenuation. PLoS ONE *7*, e37959.
29. Weiss, C., Herwig, A., and Schütz-Bosbach, S. (2011). The self in social interactions: sensory attenuation of auditory action effects is stronger in interactions with others. PLoS ONE *6*, e22723.
30. Keysers, C., Kaas, J.H., and Gazzola, V. (2010). Somatosensation in social perception. Nat. Rev. Neurosci. *11*, 417–428.
31. McGlone, F., Olausson, H., Boyle, J.A., Jones-Gotman, M., Dancer, C., Guest, S., and Essick, G. (2012). Touching and feeling: differences in pleasant touch processing between glabrous and hairy skin in humans. Eur. J. Neurosci. *35*, 1782–1788.
32. Rolls, E.T., O'Doherty, J., Kringelbach, M.L., Francis, S., Bowtell, R., and McGlone, F. (2003). Representations of pleasant and painful touch in the human orbitofrontal and cingulate cortices. Cereb. Cortex *13*, 308–317.
33. Francis, S., Rolls, E.T., Bowtell, R., McGlone, F., O'Doherty, J., Browning, A., Clare, S., and Smith, E. (1999). The representation of pleasant touch in the brain and its relationship with taste and olfactory areas. Neuroreport *10*, 453–459.
34. McCabe, C., Rolls, E.T., Bilderbeck, A., and McGlone, F. (2008). Cognitive influences on the affective representation of touch and the sight of touch in the human brain. Soc. Cogn. Affect. Neurosci. *3*, 97–108.
35. Deen, B., Pitskel, N.B., and Pelphrey, K.A. (2011). Three systems of insular functional connectivity identified with cluster analysis. Cereb. Cortex *21*, 1498–1506.
36. Kida, T., and Shinohara, K. (2013). Gentle touch activates the anterior prefrontal cortex: an NIRS study. Neurosci. Res. *76*, 76–82.
37. Essick, G.K., McGlone, F., Dancer, C., Fabricant, D., Ragin, Y., Phillips, N., Jones, T., and Guest, S. (2010). Quantitative assessment of pleasant touch. Neurosci. Biobehav. Rev. *34*, 192–203.

38. Etzi, R., Spence, C., and Gallace, A. (2014). Textures that we like to touch: an experimental study of aesthetic preferences for tactile stimuli. Conscious. Cogn. *29*, 178–188.

39. Ekman, G., Hosman, J., and Lindstroem, B. (1965). Roughness, Smoothness, and Preference: A Study of Quantitative Relations in Individual Subjects. J. Exp. Psychol. *70*, 18–26.

40. Major, D.R. (1895). On the affective tone of simple sense-impressions. Am. J. Psychol. *7*, 57–77.

41. Keverne, E.B., Martensz, N.D., and Tuite, B. (1989). Beta-endorphin concentrations in cerebrospinal fluid of monkeys are influenced by grooming relationships. Psychoneuroendocrinology *14*, 155–161.

42. Martel, F.L., Nevison, C.M., Simpson, M.J., and Keverne, E.B. (1995). Effects of opioid receptor blockade on the social behavior of rhesus monkeys living in large family groups. Dev. Psychobiol. *28*, 71–84.

43. Wittig, R.M., Crockford, C., Lehmann, J., Whitten, P.L., Seyfarth, R.M., and Cheney, D.L. (2008). Focused grooming networks and stress alleviation in wild female baboons. Horm. Behav. *54*, 170–177.

44. Dunbar, R.I. (2010). The social role of touch in humans and primates: behavioural function and neurobiological mechanisms. Neurosci. Biobehav. Rev. *34*, 260–268.

45. Silk, J.B., Alberts, S.C., and Altmann, J. (2003). Social bonds of female baboons enhance infant survival. Science *302*, 1231–1234.

Current Biology
Supplemental Information

Active Interpersonal Touch Gives Rise to the Social Softness Illusion

Antje Gentsch, Elena Panagiotopoulou, and Aikaterini Fotopoulou

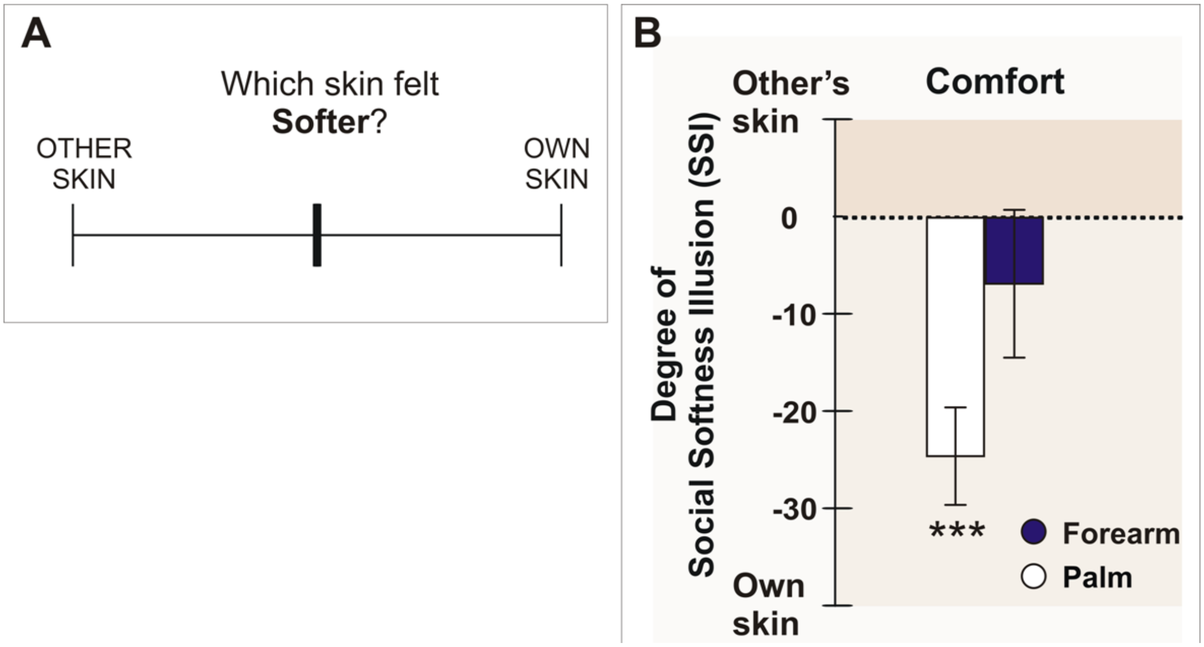
A
Which skin felt
Softer?
OTHER SKIN
OWN SKIN
B
Comfort
Other's skin
Own skin
Degree of Social Softness Illusion (SSI)
0
-10
-20
-30
***
Forearm
Palm

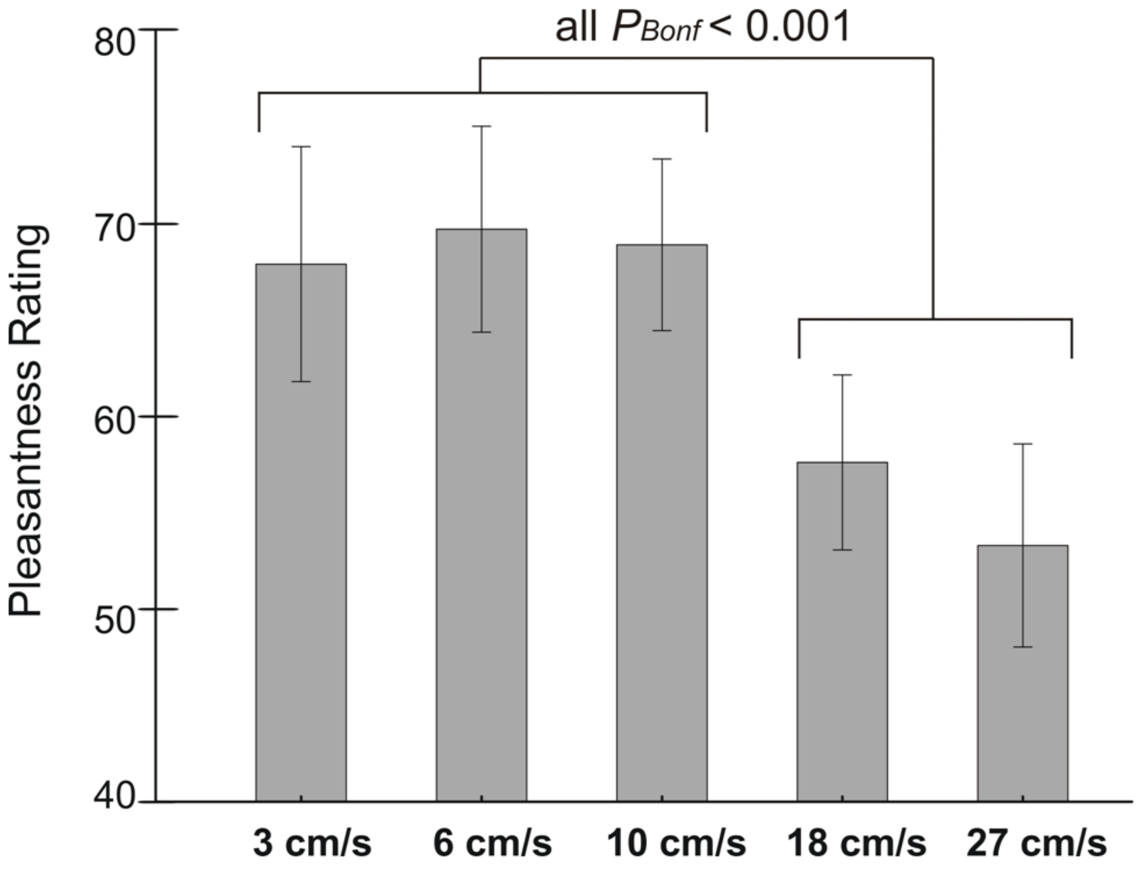
all P_{Bonf} < 0.001
80
70
60
50
40
Pleasantness Rating
3 cm/s
6 cm/s
10 cm/s
18 cm/s
27 cm/s

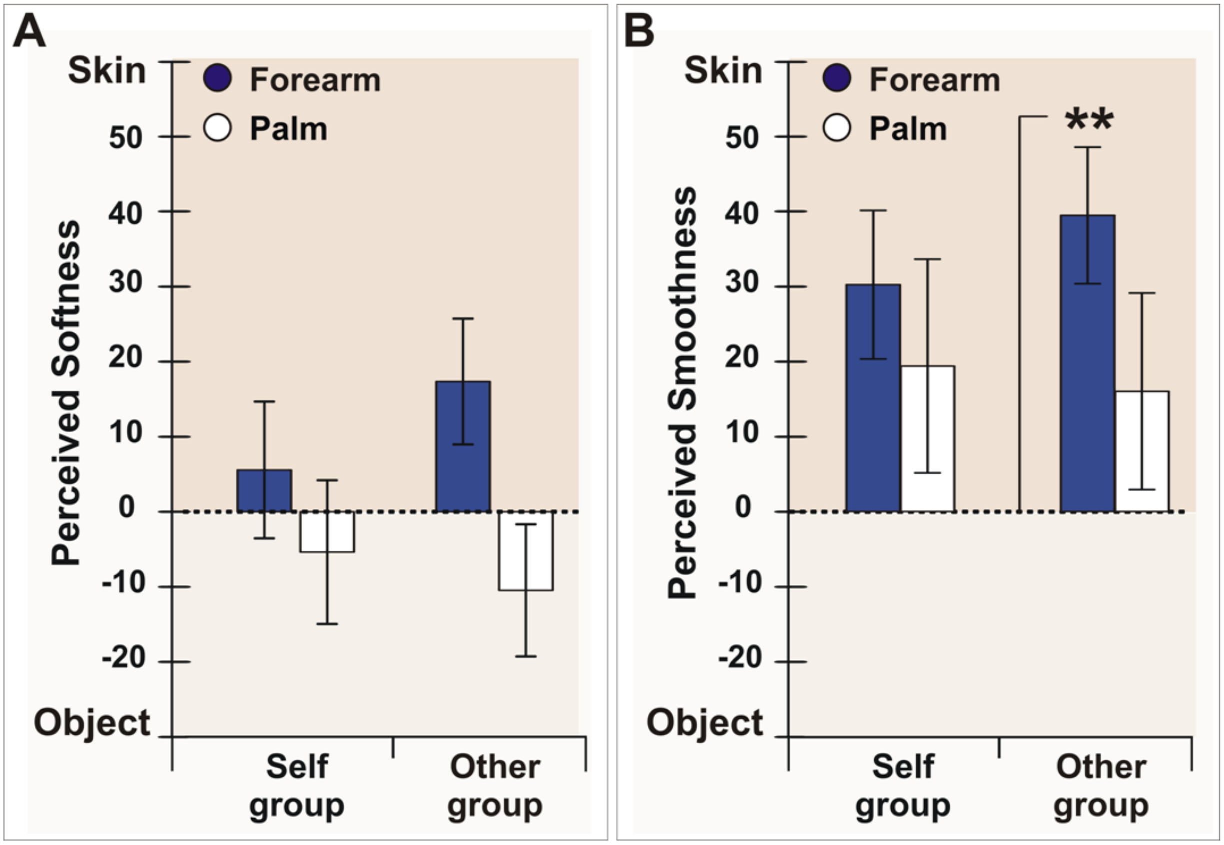
A
Skin
Forearm
Palm
50
40
30
20
10
0
-10
-20
Object
Perceived Softness
Self group
Other group
B
Skin
Forearm
Palm
**
50
40
30
20
10
0
-10
-20
Object
Perceived Smoothness
Self group
Other group

Figure S1. Visual Analogue Scale and Comfort Ratings of Experiment 1 (related to Figure 1).

(A) Example of a visual analogue scale (VAS) used for making comparative judgments between experiences during self-touch (touch of one's own skin) and other-touch (touch of the other's skin) on different perceptual attributes of the skin; the scale shown refers to softness sensations (scales were identical for other sensory and affective attributes). After each experimental trial involving self-touch and other-touch, participants were asked to indicate whether the experience of softness differed between both conditions (reflected in the dichotomous left-right judgment for other vs. self) and to which degree they felt their own skin or the other's skin to be softer (reflected in the continuous VAS score ranging from zero difference, midpoint, to an extreme difference in softness experience, endpoints). The assignment of the anchor labels, 'other skin' and 'own skin', to the two VAS endpoints was counterbalanced across participants.

(B) Comparative judgments of comfort between self-touch and other-touch are shown separately for stroking of the forearm and palm. Bars show means and standard error of the means (±SEM).

Figure S2. Results of Experiment S2 (related to Figure 2A).

Experiment S2 tested whether the stimulation velocities applied in Experiment 2 indeed modulate affective experience in response to passive touch. This additional data was obtained as part of a previous experiment (unpublished data), in which pleasantness thresholds for passive touch experience were explored across a range of stroking velocities for later use as experimental stimulation levels. A trained experimenter delivered manual strokes with a soft brush at five different velocities (3cm/s, 6cm/s, 10cm/s, 18cm/s and 27cm/s) to the forearm of 51 healthy adults. Participants were asked to verbally rate pleasantness from 0 (not at all pleasant) to 100 (very pleasant) separately for each stroking velocity (with four repetitions per velocity, applied in random order). Figure S2 shows mean pleasantness ratings with standard deviations at different stroking velocities. The results show that slow CT optimal stroking at 3 cm/s, 6 cm/s or 10 cm/s is experienced as significantly more pleasant than faster less optimal stroking at 18 cm/s or 27 cm/s (all $P_{Bonf} < 0.001$), without any difference between the slow velocities or between the fast velocities (all $P_{Bonf} > 0.69$). These results are an additional confirmation that the stimulation levels used in Experiment 3are an effective experimental manipulation of passive pleasure experience.

Figure S3. Results of Experiment S3 (related to Figure 3).

Perceptual judgments of (A) softness and (B) smoothness for touch of the forearm skin and palm skin as compared to different fabrics (see below for Experimental Procedures of Experiment 3), assessed separately in individuals touching their own skin ('Self' group, N = 12) and individuals touching another's skin ('Other' group, N = 13). Repeated measures ANOVAs calculated on mean VAS scores for each measure (softness, smoothness) with the factors Site (palm, forearm) and Group (self, other) revealed a significant main effect of Site for both softness ($F(1, 22) = 13.73$, $P < 0.01$) and smoothness ($F(1, 22) = 5.79$, $P < 0.05$). Effects involving the factor Group failed to reach significance (all $P > 0.12$). In order to specifically assess differences in the hypothesized direction of softness and smoothness judgments (i.e., the skin judged as being softer and smoother than fabric), one-sided t-tests against zero (i.e., no difference between skin and fabric) were performed on mean VAS ratings. Bonferroni correction was applied for eight comparisons (i.e., separate tests for softness and smoothness judgments of palm and forearm in both groups). In the Other-group, there was a tendency of the other's forearm being rated softer than fabrics, and the palm rated less soft than fabrics, but these tendencies did not reach statistical significance ($P_{Bonf} > 0.12$). Smoothness ratings revealed a significant effect on the forearm ($P_{Bonf} < 0.01$), indicating that the other's skin on the forearm was perceived as smoother relative to fabrics, but not on the palm ($P_{Bonf} > 0.9$). In the Self-group, there were no significant differences in the comparison of skin and fabrics regarding softness (all $P_{Bonf} > 0.59$) or smoothness (all $P_{Bonf} > 0.11$). These results provide an additional confirmation that another person's skin is experienced as smoother than one's own skin even if self and other are not compared directly but indirectly through comparison with surfaces in separate groups of individuals. Error bars represent SEM; $^{**}P < 0.01$.

Supplemental Experimental Procedures

Participants

Overall 133 right-handed healthy participants (age 23.4±4.2SD years) took part in six separate experiments for course credit or £6/hour. Participants were recruited on University College London campus, and were randomly assigned to perform the experiment in pairs. They did not know each other before the experiment. Only individuals without a history of mental, neurological and skin diseases took part. In addition, only females were included in the experiment to minimize variance in physical skin properties (such as hair coverage) and to keep the gender relation constant between participants and female experimenters. Hence, findings in this study may only be generalized to women's perception of touch. This is relevant because there is evidence for gender differences in the expression of touch [S1] and influences of female hormone levels on skin sensitivity [S2]. Further exclusion criteria were visible skin abnormalities in the target areas of hands and arms, for example, cuts, scrapes, wounds, scars or tattoos. Signed informed consent was obtained from all participants prior to their participation. The study was approved by the Ethics Committee of the Research Department of Clinical, Educational and Health Psychology, University College London.

General Experimental Set-Up

Participants in each experiment were randomly paired to arrive at the laboratory at the same time and to perform reciprocal touch on each other, thus assuming both the role of the touch giver ('active participant') and the touch recipient ('passive participant'), in succession (see also main experimental procedures below). They were told the experiment was about the perception of tactile sensations. Upon arrival, participants were assigned a seat in the room next

to each other (30 cm apart) at the same side of a table, however, facing opposite directions. That is, the active participant was facing the computer screen on the table, and the passive participant was sitting on her left-hand side with the back to the table (see Figure 1A, main text). This set-up was chosen to provide a flexible possibility for both participants to comfortably rest their left forearms on the table by aligning them parallel to each other in a 45 degree angle relative to the active participant's body. A curtain was installed between participants to prevent visual contact between them, and the passive participant reached underneath it to place her arm in front of the active' participant. The position of the arm facing upwards allowed access to both hairy skin (forearm) and glabrous skin (palm) while maintaining constant arm position across experimental trials. The right arm (i.e., the stroking hand) of the active participant was resting on the table in front of her between trials, while the passive participant's right arm was out of view.

The Touch Protocol

Prior to each of the main experiments, each active participant was trained individually according to an experimental 'touch protocol'. Equal areas of nine cm length and four cm width were marked on the volar side of the forearm and the palm of the active and passive participant, which indicated the exact site of touch. The forearm and palm are commonly used body sites in studies investigating the function of the C-tactile (CT) afferent system [S3, S4, S5], as CT afferents are found exclusively in hairy skin surfaces [e.g., arm: S6, S7] but not in the glabrous skin [e.g., palm: S8, S9]. Active participants were instructed to press lightly with three fingers of the right hand (index, middle and ring finger) against their own left arm skin ('self-touch') or the passive participant's left arm skin ('other-touch') and perform dynamic stroking movements within the marked skin area in a distal to proximal, linear direction. In experiment 2 and 3, only two fingers of the right hand (index and middle finger) were used, which allowed more precise

control of the finger-skin contact area for manipulations of stroking speed (involving precise instructions of onset and offset of finger-skin contact) or touch of external objects. The stroking movement was done at a slow speed unless the speed of stroking was explicitly manipulated (see experiment 2). Specifically, participants were instructed to cover the marked skin area (of nine cm length) within a 3 second time interval, resulting in a stroking velocity of approximately 3cm/sec. Indentation forces of < 0.3 N were targeted, which was achieved by the experimenter demonstrating a finger weight of < 100gr (as determined by a digital weight scale). Specifically, during practice trials, the experimenter demonstrated the target type of finger-skin contact on participants forearm, and participants were instructed to match the touch on the experimenter's forearm. Participants were also trained to deliver the movements in synchrony with computerized visual cues (i.e., "touch now" appeared on the screen; see experiment 1, 3-5), auditory cues (i.e., sounds; see experiment 2), or followed the instructions of the experimenter (i.e., "touch now", see experiment 6). Computer-generated stimulation was controlled by a customized software program (Presentation software, Neurobehavioral Systems Inc.) and presented on a desktop computer placed at a viewing distance of approximately 80 cm. The order of self-touch and other-touch was instructed by the experimenter at the beginning of each trial. The experimenter did not proceed to the main experiment unless she felt that the active participant was able to follow this touch protocol consistently.

Main Experimental Procedures

During the main part of each experiment, each touch trial lasted 12 seconds and consisted of self-touch (repeated twice successively, for a total duration of six seconds) and other-touch (repeated twice successively, for a total duration of six seconds). The order of self-touch and other-touch (i.e., self-other, other-self) events was randomized across trials in all experiments.

To account for the potential role of vision, the availability of visual information of the touched skin area differed between experiments. In some experiments, the active participant was able to see both arms (experiment 1, 4), while in others participants were blindfolded (experiment 2, 6) or a wooden board mounted above the table prevented participants from seeing their arms which were placed underneath (experiment 3, 5). In conditions without vision, participants were guided by the experimenter to the marked skin area (experiment 2, 5, 6) or were trained on all locations at the beginning of the testing session (experiment 3), and then did the stroking movement on their own.

After each touch trial, the active participant was asked to compare self-touch versus other-touch on two main sensory dimensions of tactile perception, namely softness-hardness, roughness-smoothness, and one affective dimension, namely comfortable-uncomfortable (see below for selection of these labels). The order of the dimensions was counterbalanced across trials. Judgments were made using a computerized continuous visual analogue scale (VAS, see Figure S1). For each sensory and affective dimension, a 200 mm horizontal VAS line appeared in the centre of the computer screen with a midpoint anchor and two anchor labels at each end (e.g., 'other skin' on the left and 'own skin' on the right end, counterbalanced across participants). Participants placed a mouse cursor at a selected location on the line and confirmed with a mouse click. The cursor position was converted into a numerical VAS score from -100 to 100, where zero is no perceived difference between self- and other-touch, and ratings deviating from zero being indicative of the degree to which a specific tactile sensation occurred during self-touch or other-touch.

Participants were instructed not to communicate throughout the experiment. The experimenter monitored task and touch protocol compliance throughout the whole session. After

completion of the experiment, participants reversed active and passive participant roles, such that the previously passive participant repeated the whole experimental procedure and actively touched the previously active participant. This pairing and role reversal strategy controlled for any actual individual differences in physical skin properties between participants as they performed perceptual judgments of each other's skin. To prevent systematic order effects, participants were randomly assigned to the roles at the beginning of the experiment. Moreover, the role of the order of these participant roles was explored statistically (see below).

Dependent Measures and Statistical Analysis

Contrary to the plethora of studies investigating the relation between tactile stimulation parameters and discriminatory aspects of touch, there is a scarcity of data regarding such stimulus parameters and affective aspects of touch. Thus, the perceptual effects of parameters such as friction and roughness on affective touch remain poorly understood. Moreover, most of the existing studies have looked at fabric perception, finding for example that fabrics rated as soft and smooth are also perceived as pleasant [S3]. Such studies may miss important aspects of skin-to-skin contact such as arousal, warmth, or comfort. Given that the present study focused on skin-to skin contact, we first conducted a pilot study (N = 22) to determine our dependent measures and particularly the verbal labels that may optimally capture differences between self-touch and other-touch on the skin. Participants were asked to make comparative judgments between both touch conditions with respect to relevant sensory dimensions (such as hard-soft, rough-smooth), affective properties (such as comfort, pleasure, arousal) and of a number of other control items (such as warmth, numbness) on the above visual analogue scales. These attributes have been previously shown to represent important and salient dimensions of tactile texture perception [see also, S5, S10, S11, S12, S13]. Descriptive data showed that compared to self-

touch, touching someone else's skin yielded higher ratings for softness and smoothness (M=43.27 vs. M=32.17; M=44.40 vs. M=30.20), but lower ratings for comfort (M=15.48 vs. M=30.82). None of the other items displayed any difference at the descriptive level, possibly lacking specificity or discrete meaning in this context. Indeed, qualitative feedback from participants and the opposite direction of results between softness/smoothness and comfort, suggested that verbal labels like 'pleasantness' and 'arousal' may be sub-optimal for skin-to-skin touch as they can be interpreted to include more than one of the above meanings (e.g. touching someone's else's skin may feel both pleasant in its 'softness' but unpleasant in its 'unfamiliarity'). Thus, three labels-dimensions were selected for testing in the main experiments, namely softness, smoothness and comfort.

Statistical differences between the critical within-subjects, experimental factors in each of the experiments below were assessed separately for these three selected dimensions of tactile perception (captured by the three VAS ratings following each touch trial, see procedures above) by means of repeated-measures analysis of variance (ANOVA). Post-hoc tests were performed to examine the direction of main and interaction effects. In order to specifically assess whether participants tended to attribute one of the relevant sensory dimension more towards their own or the other's skin one-sided t-tests against zero (i.e., no difference between self and other) were performed on the VAS ratings as the dependent variables. When repeated statistical tests were conducted to evaluate such biases for different VAS ratings within the same experiment, a multiple-comparison correction was used. This correction was done by applying the Bonferroni (Holm) sequential procedure, and the Bonferroni adjusted p-values are reported as P_{Bonf}. To test for order effects of participants roles (i.e., whether assuming the active or passive role first within a pair of participants), a separate repeated measures ANOVA was conducted in each

experiment with participant order as a between-subject factor and the relevant VAS rating as the dependent variable.

Details of Experimental Procedures of Experiments 1 to 6

Experiment 1: In a first experiment (N=28, age 22.6±2.0 years, all right-handed females), we assessed whether other-touch would be associated with more positive sensory experiences than self-touch, particularly in hairy skin, as CT afferents that have been associated with greater tactile pleasure, are found in hairy, but not in glabrous (hairless) skin [S14, S15]. Participants were asked to touch the skin of the palm (glabrous skin) or the forearm (hairy skin), and compare sensations of softness, smoothness and comfort during self-touch versus other-touch. Two equal areas of nine cm were marked on the volar side of the forearm and the palm, and the touch was performed with full vision. Participants synchronized their stroking movements with computerized visual cues, resulting in a stroking velocity of approximately 3cm/sec, in accordance with the main touch protocol and experimental procedures (as described above). Each site of touch (i.e., palm and forearm) was repeated twice in both orders (i.e., self-other, other-self), for a total of eight 12 second trials. After each trial, participants were asked to make comparative judgments between self and other on the above three, separate VAS scales for softness, smoothness and comfort. The entire session took approximately 30 minutes. Questions during debriefing revealed that none of the participants had consciously experienced any systematic differences between their own and other people's skin before taking part in the experiment. However, after the experiment some of the participants did spontaneously report the feeling that the other participant's skin was softer than their own.

Experiment 2: Thirteen pairs of naïve participants (N=26, age 22.2±2.9 years, all right-handed females) were recruited for experiment 2 in order to further systematically investigate the

emotional significance of interpersonal touch as a possible mediator of the phenomenon observed in experiment 1. The emotional significance of touch was experimentally manipulated based on insights about the fundamental psychophysical properties of the neurophysiological system that sustains passive, affective touch. In particular, one well-established property is that C-tactile afferents are optimally stimulated by tactile stimulation speeds in the range from 1-10 cm/s (see main text). Participants performed different types of touch movements of increasing velocity on the skin during both conditions of self-touch and other-touch. Each trial consisted of either static touch or lateral motion at 3 cm/s, 10 cm/s, or 18 cm/s. Psychophysical data has shown that touch at 3 cm/s or 10 cm/s is perceive as more pleasant than touch at 18 cm/s [S3, e.g., S16]. Moreover, while stroking movements at 3 cm/s and 10 cm/s velocities are known to be optimal for CT afferent activation, faster touch and slower touch is less optimal in eliciting CT activity [S14].

These different types of movements were performed under conditions of self-touch and other-touch on the palm and forearm. Prior to the experimental trials, participants were trained to deliver the stroking movements in synchrony with computer-generated tones. During each touch trial, six tones were presented at a rate of one per second (see Figure S2). The first set of three tones signaled self-touch, followed by a pause of two seconds and a second set of three tones which signaled other-touch. During each set of three tones, the participant’s task was to stroke the skin in synchrony with every tone by covering a skin area of nine cm in distal to proximal direction and by using the index and middle fingers of the right hand (see also main touch protocol). In the slow speed condition (3 cm/s), they slowly performed one stroking movement across the whole area within three seconds (i.e., for the duration of all three tones). In the medium speed condition (10 cm/s), they performed three stroking movements within three

seconds (i.e., one stroke at each tone), and in the high speed condition (18 cm/s) they performed six stroking movements within three seconds (i.e., covering the complete area twice at each tone). During the static touch condition, participants kept gentle skin contact without relative motion for the duration of all three tones. After each touch, comparative judgments between self-touch and other-touch were obtained for softness sensations by means of a VAS scale as in the first experiment (see also main procedures). The four touch conditions were presented in blocks (in random order between participants), and each trial was repeated four times for each body site, for a total of 32 trials. Throughout the experiment, participants performed the touch without vision (blindfolded).

Experiment 3: In a third experiment, we investigated whether the feeling of greater softness and smoothness for the other's skin can also be indirectly measured by avoiding direct self-other comparison and thereby minimizing potential biases in attention, stroking, or responding (such as social desirability bias). Nine, new pairs of naïve female participants (N=18, age 24.8±6.9 years, all right-handed females) were recruited for this experiment. Participants were asked to compare their own and the other participant's skin relative to external reference objects. These objects consisted of three rectangular pads (made of felt) of one cm in height and four cm in width and nine cm in length, which were covered by three different fabrics. The three fabrics were made of cotton, fleece and fur materials. Participants were seated next to each other with their arms placed in parallel on a table (as described in the main procedure), and a wooden board was mounted above the table (about 30 cm height) to prevent participants from seeing their arms and the fabrics. The fabrics were placed one at a time underneath the board on the table in front of the active participant and in equal distance from the hands of both participants (about five cm distance), and the touch was performed without vision. The spatial positions of

hands and object were marked on the table and visible only to the experimenter (sitting opposite the table) who placed the fabrics in the same location throughout the experiment to keep spatial relations constant. Participants were trained to locate the other participant's arm as well as the fabric-covered object at the beginning of the testing session.

Before the main experiment, participants were asked to judge the softness of the three fabrics separately on a VAS scale requesting an intensity rating (from 'very rough' to 'very soft'). Subsequently, in the main experiment, participants compared each fabric with their own skin or the other participant's skin in separate blocks. The touch was performed by alternating between skin-touch and fabric-touch, and touch of the skin was performed at two different body sites, forearm and palm (in separate trials). For each body site, each of the three fabrics was presented four times, resulting in a total of 24 trials of self-touch and 24 trials of other-touch. Experimental blocks of self-touch and other-touch were counterbalanced across participants, and the sequence of conditions within each block was randomized. One experimental trial lasted 12 seconds, and participants delivered the touch in synchrony with computerized visual cues (as described in the main procedure). After each trial, participants made comparative judgments concerning softness sensations using a similar VAS scale as in the experiments before, however, the two anchor labels now indicated 'skin' and 'object' (the assignment of the labels to left and right endpoints of the scale was counterbalanced across participants). That is, while in experiment 1 and 2 participants made direct self-other comparisons, in experiment 3 they made direct skin-object comparisons.

Experiment 4: Eighteen pairs of naïve participants (N=36, age 23.8±3.9 years, all right-handed females) were recruited to test the potential role of spatial proximity in the SSI. The results of experiment 1 and 2 suggested that the illusion of feeling greater softness for someone

else's skin depends on the emotional significance of touch associated with the stimulation of CT afferents that are known to innervate hairy skin. This interpretation is in line with experiment 1 in which the SSI only appeared for touch of the forearm (hairy skin) but not the palm (glabrous skin). However, as the forearm is more proximal than the palm, the contribution of spatial proximity and related salience effects cannot be ruled out. Therefore, experiment 4 tested the hypothesis that if spatial proximity does not contribute to the SSI, touch on both proximal and distal locations of self and other forearm should show the illusion. Participants were asked to perform touch at proximal and distal locations of the forearm on self and other, and comparative judgments concerning softness, smoothness and comfort were obtained after each comparison (same procedure as in experiment 1). Two equal areas of nine cm were marked on the volar side of the lower and upper part of the forearms of both participants indicating the exact site of touch. The touch was performed with full vision and in accordance with the touch protocol and experimental procedures, as described above. Each of the two sites of touch (i.e., upper and lower forearm) was repeated twice in both orders (i.e., self-other, other-self), for a total of eight trials. Participants delivered the movements in synchrony with computerized visual cues, resulting in a stroking velocity of approximately 3 cm/sec (see main procedures).

Experiment 5: The results of experiment 3 and 4 suggested that the SSI is unlikely to result from more general top-down response biases. Experiment 5 aimed at further understanding its specificity with respect to bottom-up sensory signals and top-down social factors, by testing whether similar self-touch and other-touch judgments would lead to the SSI, when there was no availability of sensory information from direct skin contact. Thirteen pairs of naïve female participants (N=26, age 22.2±2.8 years, all right-handed females) were recruited for this experiment and asked to compare the effects of self-touch and other-touch on fabrics attached to

the palm or forearm of another person and to their own. Fabrics were attached to both (active and passive) participants' forearms such that the skin was fully covered and self-touch and other-touch was performed as described in the Main Experimental Procedures (see above), however without direct skin contact. Identical pieces of cotton fabric were wrapped around the forearm and palm of active and passive participant and they both were blindfolded during the procedure of attaching the fabrics to exclude influences of prior visual information. The velocity of touch and the extent of movement was demonstrated and monitored throughout the session by the experimenter. During the touch participants were not blindfolded, but a wooden board mounted above the table (as in experiment 3) prevented participants from seeing their arms which were placed underneath the table. The touch was performed on three different sites of touch, upper and lower forearm and palm, in random order with four trials per condition, resulting in a total of 12 trials. The stroking movement was guided by computerized visual instructions and performed in accordance with the touch protocol and experimental procedures, as described above. Again, participants were asked to make several comparative judgments between self-touch and other-touch (as before), however, this time referring to properties of the fabrics attached to their own versus the other participant's skin. After each touch trial the same VAS rating appeared as in experiment 1 (see also main procedure), however, instead of the labels 'other skin' and 'own skin' the anchor labels were changed into 'own fabric' and 'other fabric'.

Experiment 6: A sample of 11 pairs of participants (N=22, age 24.1±3.9 years, all right-handed females) took part in experiment 6 which aimed at investigating whether the observed illusion is the result of predictive mechanism related to the voluntary control of action and leading to sensory self-attenuation (see main text). To test this hypothesis, the degree of voluntary control over the stroking movement was varied. Participants were asked to rate the

skin softness while they were actively or passively touching their own skin or the other participant's skin on the forearm. In a passive movement condition, the movements were fully controlled by the experimenter who manually guided the participant's right hand. In two active touch conditions, participant's performed the touch either alone (individual active touch) or was joint by the experimenter (joint active touch) who touched the participant's hand during the movement however without interfering or guiding the hand as in the passive movement condition. That is, in both active touch conditions, participants fully controlled (i.e., initiated and executed) the movement themselves. The joint active touch condition served to control for potential influences of attentional distraction on perceptual judgments in the passive touch condition due to the presence of the experimenter and tactile sensations during guidance of the hand. The three action conditions were tested in separate blocks (order counterbalanced across participants), with 4 trials per action condition for a total of 12 trials. Participants were blindfolded during the touch and alternated between self-touch and other-touch within each trial. The touch trials were practiced and performed in accordance with the touch protocol and experimental procedures, as described above.

Supplemental Analysis of Experiments 1 to 6

Experiment 1: A repeated measures ANOVA was calculated separately on the average VAS scores for each measure (comfort, softness, smoothness) with the factor Site (two levels: palm, forearm) as a within-subject factor. We found a significant main effect of Site for both perceived softness ($F(1, 27) = 9.34$, $P < 0.01$) and smoothness ($F(1, 27) = 6.79$, $P < 0.05$). Moreover, a significant effect of Site on perceived comfort was found ($F(1, 27) = 6.08$, $P < 0.05$). In order to test for the direction of these effects, we conducted further post-hoc one-sided t-tests (means against zero difference between self and other), which were Bonferroni corrected

for six comparisons (i.e., three dependent measures at two body sites). These post-hoc tests showed that participants significantly judged the other's forearm skin to be softer (t = -4.00; P_{Bonf} = 0.002) and smoother (t = -3.41; P_{Bonf} = 0.008) than their own forearm skin (see Figure 1B, main text). For the palm, no difference in experienced skin softness (P_{Bonf} > 0.9) or smoothness (P_{Bonf} = 0.76) was found. For touch of the palm, however, other-touch was experienced as less comfortable than self-touch (t = 4.87; P_{Bonf} < 0.001), but this difference was not significant for the forearm (P_{Bonf} > 0.9; see also experiment 3). Separate repeated measures ANOVAs testing for order effects of participants roles (see experimental procedures) revealed no significant effect on any type of VAS rating, softness (P = 0.39), smoothness (P = 0.52) or comfort (P = 0.17).

Experiment 2: A repeated-measures ANOVA with two factors Site (two levels: palm, forearm) and Velocity (four levels: 3 cm/s, 10 cm/s, 18 cm/s, static touch) was calculated on softness ratings. Results revealed a significant main effect of Velocity ($F(3, 81) = 3.08$, P = 0.03), but no significant main effect or interaction effect involving the factor Site (all *P-values* > 0.59). Subsequently, the VAS ratings were averaged across both sites and the direction of the main effect of Velocity was tested by means of post-hoc one-sided t-tests (means against zero difference between self and other) which were Bonferroni corrected for four comparisons (i.e., four velocities). The SSI appeared most strongly during 3 cm/sec (t = -2.74; P_{Bonf} = 0.03) and 10 cm/sec (t = -3.69; P_{Bonf} = 0.004; see Figure 2A), but was absent during 18 cm/sec touch (P_{Bonf} > 0.9) or static touch (P_{Bonf} = 0.77). A repeated measures ANOVA testing for general order effects of participants roles (see experimental procedures) revealed no significant effect on softness ratings (P = 0.44).

Experiment 3: Judgements of softness of the three fabrics separately indicated that perceived softness differed significantly between the fabrics, as revealed by a repeated measures

ANOVA main effect of Fabric (cotton, fleece, fur; ($F(2, 34) = 54.55$, $P < 0.001$). Post-hoc Bonferroni tests (corrected for three comparisons) indicated that all three fabrics differed significantly from each other (all *Ps* < 0.001), with fur receiving the highest softness ratings (M=73.74), followed by fleece (M=34.37), and cotton being perceived as neutral to slightly rough (M=-10.95). This confirmed the choice of materials for manipulating stimulus softness. To examine the direction of effects on the rough-soft dimension, one-sided t-tests (means against zero, indicating neither rough nor soft) were performed, Bonferroni corrected for three tests (i.e., the three Fabric conditions). The results showed that fur and fleece were judged to be soft (all $P_{Bonf} < 0.001$), while cotton was judged to be neither rough nor soft ($P_{Bonf} = 0.21$).

A repeated measures ANOVA with the three factors Fabric (three levels: fur, fleece, cotton), Site (two levels: forearm, palm), and Self-Other (two levels: self-touch, other-touch) was calculated on softness ratings. This analysis revealed three significant main effects, without any significant interaction between them (all *P*s > 0.37). The significant main effect of Fabric ($F(2, 34) = 27.02$, $P < 0.001$) confirmed the difference in perceived softness between materials. A main effect of Site ($F(1, 17) = 8.69$, $P = 0.01$) indicated that the forearm skin was generally perceived as softer than the palm relative to fabrics attached to an external object. The main effect of Self-Other ($F(1, 17) = 10.95$, $P = 0.004$) indicated that, relative to fabrics of varying softness, the other's skin (other-touch) was judged as being on average softer than one's own skin (self-touch). In order to specifically assess whether participants tended to experience more softness during touch of the skin or touch of the fabric one-sided t-tests against zero (i.e., no difference between skin and fabric) were performed on the mean VAS ratings (averaged across the fabrics), separately for the different conditions of the experimental factors Site and Self-Other. These post-hoc tests were Bonferroni corrected for four comparisons (i.e., separate tests

conducted for self- and other-touch on palm and forearm). The results showed that other-touch on the forearm was rated to be softer compared to the fabrics on average (t = -3.11; P_{Bonf} = 0.02). By contrast, other-touch on the palm or self-touch on forearm and palm was rated to be equally soft compared to the fabrics (all P_{Bonf} > 0.19). A separate repeated measures ANOVA testing for order effects of participants roles (see experimental procedures) revealed no significant effect on softness ratings (P = 0.98), and there was no significant interaction between order and any of the three experimental factors (i.e., Fabric, Site and Self-Other; all Ps > 0.33).

Experiment 4: A repeated measures ANOVA was calculated with the factor Site (two levels: proximal, distal) as a within-subject factor. No significant main effect of Site was revealed for perceived softness (P = 0.83), smoothness (P = 0.87) or comfort (P = 0.67). One-sided t-tests (means against zero difference between self and other) were performed, Bonferroni corrected for six comparisons (i.e., three dependent measures at two body sites). These tests showed that the SSI was present at both for proximal touch (t = -2.91; P_{Bonf} = 0.02; touch of the upper forearm) and distal touch (t = -3.00; P_{Bonf} = 0.02; touch of the lower forearm). Similar results were obtained for smoothness ratings for upper forearm (t = -3.25; P_{Bonf} = 0.01) and lower forearm (t = -3.09; P_{Bonf} = 0.02). No significant effect for comfort ratings was revealed on the upper forearm (P_{Bonf} = 0.24) or lower forearm (P_{Bonf} = 0.19). Separate repeated measures ANOVAs testing for order effects of participant roles (see experimental procedures) revealed no significant effect on any type of VAS rating, softness (P = 0.51), smoothness (P = 0.96) or comfort ratings (P = 0.69).

Experiment 5: A repeated measures ANOVA was calculated with the factor Site (three levels: upper forearm, lower forearm, palm) as a within-subject factor. The main effect of Site on perceived softness did not reach significance (F(2, 50) = 2.84, P = 0.07), and no main effect of

Site was present for perceived smoothness ($P = 0.39$) or comfort ($P = 0.89$). For softness ratings at the descriptive level, the SSI appeared to be reversed for the upper and lower forearm and appeared to emerge for the palm. To test for the significance of these observations, one-sided-t-tests were performed, Bonferroni corrected for three tests (i.e., three different body sites). These tests revealed that the SSI was not present at any of the three sites of touch (upper forearm, $P_{Bonf} = 0.54$; lower forearm, $P_{Bonf} = 0.39$; palm $P_{Bonf} = 0.14$). A separate repeated measures ANOVA testing for order effects of participants roles (see experimental procedures) revealed no significant effect of order on ratings of fabric softness ($P = 0.52$).

<u>*Experiment 6*</u>: A repeated measures ANOVA with the factor Action (three levels: individual, joint, passive) showed a significant main effect on softness ratings ($F(2, 42) = 12.66$, $P < 0.001$; see Figure 2B). Bonferroni-corrected post-hoc comparisons revealed that the passive action condition differed significantly from the individual action condition ($P_{Bonf} < 0.001$) and the joint action condition ($P_{Bonf} = 0.01$), without a difference between the latter two ($P_{Bonf} = 0.27$). Further one-sided t-tests (means against zero difference between self and other) tested for the direction of these effects and were Bonferroni corrected for three tests (i.e., the three Action conditions). These tests showed that the SSI was present for both individual actions ($t = -3.77$; $P_{Bonf} = 0.003$) and joint actions ($t = -2.89$; $P_{Bonf} = 0.02$) but disappeared in the passive action condition ($P_{Bonf} = 0.54$). There was no significant effect of order of participant roles (see experimental procedures) on softness ratings ($P > 0.69$). Moreover, the experimental action conditions did not differ significantly with respect to the reported comfort experience ($P = 0.17$), according to a separate repeated measures ANOVA performed on comfort ratings.

Supplemental Discussion

<u>*Multisensory Effects*</u>

Given that some but not all of our experiments involved an interplay between vision and tactile processing, the SSI could be accounted for by multisensory integration effects. Such an interpretation would rely on observations of cross-modal influences taking place at early levels of sensory-specific processing [S17], and research indicating the involvement of primary somatosensory cortex (S1) even when touch is merely observed [S18]. The large size of the SSI in Experiment 1 could be explained by the availability of visual input, which was controlled for example by blindfolding participants in Experiment 2, in which a much smaller SSI was observed. One might speculate that simply viewing the body part may facilitate simulation mechanisms and thereby lead to an increase in self-other differentiation in tactile perception. Indeed, a recent study demonstrated that observed touch directed to another person's hand elicits spatially similar brain activations, and to a larger degree, than when touch of one's own hand is observed [S19]. Moreover, viewing CT-optimal touch (i.e., observed caress) gives rise to a similar response in the posterior insula as directly felt caressing touch [S20]. Given the pattern of results and specificity of the SSI across experiments, however, it is very unlikely that visual influences alone or general attentional differences between self-touch and other-touch can account for the SSI. Instead, in our study, the illusion varied most strongly with the affective significance of the touch (Experiment 2) and the presence of motor control signals (Experiment 6). Moreover, apart from a lack of visual input, the reduced size of the SSI in Experiment 2 could also be explained by the additional auditory input (i.e., simultaneous touch guidance) and increased task difficulty (i.e., different stroking speeds). Taken together, even though the SSI may be potentially enhanced by vision, it cannot be explained by multisensory effects alone.

Comfort Ratings and Touch of the Palm

Comfort experience is a relevant dimension of social touch, and touching a stranger is typically accompanied by a normal feeling of uncomfortableness as compared to self-touch. Surprisingly, in Experiment 1, this tendency was found only for touch of the palm but not for the forearm which was rated as being equally comfortable to touch for self and other. One possible explanation of these results might be the simultaneous focus on rewarding tactile dimensions of the haptic experience of skin texture such as softness and smoothness which may have influenced comfort ratings. Comfort experience is a dimension, similar to pleasure, which can be composed of more than one meaning, from social contextual cues to basic haptic sensations. Hence, the experienced haptic illusion of increased softness of another's skin may have overruled the normal uncomfortableness induced by the social context of touching the forearm of an unfamiliar other person. This was not the case for touch of the palm for which the softness illusion was absent.

It should also be noted that the site specificity of the SSI showed some meaningful variability across experiments. While significant differences between body sites (palm vs. forearm) were found in Experiment 1 and 3, no significant interactions involving the site of touch were observed in Experiment 2 (please also see above regarding the more general and multisensory integration reasons regarding the smaller SSI effects in this experiment). The SSI appeared in Experiment 2 not only for touch of the forearm but also the palm, but only when the touch was performed at stroking speeds of 3 cm/s or 10 cm/s that are optimal for stimulation of slow-conducting unmyelinated C-tactile afferents [S6, S7, S21]. CT afferents innervating the hairy skin of the body have been shown to convey cutaneous sensations to brain structures related to emotion [S6, S7, S15] and thereby contribute to hedonic ratings [S14]. The palm is a glabrous skin site which is devoid of CT afferent innervations [S22, S23], however, previous

studies investigating passive touch of the palm have found that CT optimal stroking of the palm increases pleasantness ratings in comparison to non-CT optimal velocities [S4, S24] and such ratings can be affected by previous touch on CT-innervated hairy skin [S25]. These effects point to the importance of learned experiences associated with touch in CT optimal speeds even when one is touched on the palm. Thus, it seems likely that similar top-down influences based on learned experiences of touch pleasure may contribute to the SSI, over and above the actual, bottom-up activation of the CT afferent system.

Supplemental References

S1. Hertenstein, M.J., and Keltner, D. (2011). Gender and the Communication of Emotion Via Touch. Sex Roles *64*, 70-80.

S2. Gallace, A., and Spence, C. (2014). In touch with the future: The sense of touch from cognitive neuroscience to virtual reality (Oxford, UK: Oxford University Press).

S3. Essick, G.K., McGlone, F., Dancer, C., Fabricant, D., Ragin, Y., Phillips, N., Jones, T., and Guest, S. (2010). Quantitative assessment of pleasant touch. Neurosci Biobehav Rev *34*, 192-203.

S4. McGlone, F., Olausson, H., Boyle, J.A., Jones-Gotman, M., Dancer, C., Guest, S., and Essick, G. (2012). Touching and feeling: differences in pleasant touch processing between glabrous and hairy skin in humans. Eur J Neurosci *35*, 1782-1788.

S5. Guest, S., Essick, G., Dessirier, J.M., Blot, K., Lopetcharat, K., and McGlone, F. (2009). Sensory and affective judgments of skin during inter- and intrapersonal touch. Acta Psychol (Amst) *130*, 115-126.

S6. Vallbo, A., Olausson, H., Wessberg, J., and Norrsell, U. (1993). A system of unmyelinated afferents for innocuous mechanoreception in the human skin. Brain Res *628*, 301-304.

S7. Vallbo, A.B., Olausson, H., and Wessberg, J. (1999). Unmyelinated afferents constitute a second system coding tactile stimuli of the human hairy skin. J Neurophysiol *81*, 2753-2763.

S8. Olausson, H., Wessberg, J., Morrison, I., McGlone, F., and Vallbo, A. (2010). The neurophysiology of unmyelinated tactile afferents. Neurosci Biobehav Rev *34*, 185-191.

S9. Johnson, K.O., Yoshioka, T., and Vega-Bermudez, F. (2000). Tactile functions of mechanoreceptive afferents innervating the hand. J Clin Neurophysiol *17*, 539-558.

S10. Hollins, M., Faldowski, R., Rao, S., and Young, F. (1993). Perceptual dimensions of tactile surface texture: a multidimensional scaling analysis. Percept Psychophys *54*, 697-705.

S11. Hollins, M., Bensmaia, S., Karlof, K., and Young, F. (2000). Individual differences in perceptual space for tactile textures: evidence from multidimensional scaling. Percept Psychophys *62*, 1534-1544.

S12. Picard, D., Dacremont, C., Valentin, D., and Giboreau, A. (2003). Perceptual dimensions of tactile textures. Acta Psychol (Amst) *114*, 165-184.

S13. Guest, S., Dessirier, J.M., Mehrabyan, A., McGlone, F., Essick, G., Gescheider, G., Fontana, A., Xiong, R., Ackerley, R., and Blot, K. (2011). The development and

validation of sensory and emotional scales of touch perception. Atten Percept Psychophys *73*, 531-550.

S14. Loken, L.S., Wessberg, J., Morrison, I., McGlone, F., and Olausson, H. (2009). Coding of pleasant touch by unmyelinated afferents in humans. Nat Neurosci *12*, 547-548.

S15. Olausson, H., Lamarre, Y., Backlund, H., Morin, C., Wallin, B.G., Starck, G., Ekholm, S., Strigo, I., Worsley, K., Vallbo, A.B., and Bushnell, M.C. (2002). Unmyelinated tactile afferents signal touch and project to insular cortex. Nat Neurosci *5*, 900-904.

S16. Crucianelli, L., Metcalf, N.K., Fotopoulou, A.K., and Jenkinson, P.M. (2013). Bodily pleasure matters: velocity of touch modulates body ownership during the rubber hand illusion. Front Psychol *4*, 703.

S17. Driver, J., and Noesselt, T. (2008). Multisensory interplay reveals crossmodal influences on 'sensory-specific' brain regions, neural responses, and judgments. Neuron *57*, 11-23.

S18. Blakemore, S.J., Bristow, D., Bird, G., Frith, C., and Ward, J. (2005). Somatosensory activations during the observation of touch and a case of vision-touch synaesthesia. Brain *128*, 1571-1583.

S19. Kuehn, E., Trampel, R., Mueller, K., Turner, R., and Schutz-Bosbach, S. (2013). Judging roughness by sight--a 7-Tesla fMRI study on responsivity of the primary somatosensory cortex during observed touch of self and others. Hum Brain Mapp *34*, 1882-1895.

S20. Morrison, I., Bjornsdotter, M., and Olausson, H. (2011). Vicarious responses to social touch in posterior insular cortex are tuned to pleasant caressing speeds. J Neurosci *31*, 9554-9562.

S21. Wessberg, J., Olausson, H., Fernstrom, K.W., and Vallbo, A.B. (2003). Receptive field properties of unmyelinated tactile afferents in the human skin. J Neurophysiol *89*, 1567-1575.

S22. Johansson, R.S., and Vallbo, A.B. (1979). Tactile sensibility in the human hand: relative and absolute densities of four types of mechanoreceptive units in glabrous skin. J Physiol *286*, 283-300.

S23. Kakuda, N. (1992). Conduction velocity of low-threshold mechanoreceptive afferent fibers in the glabrous and hairy skin of human hands measured with microneurography and spike-triggered averaging. Neurosci Res *15*, 179-188.

S24. Ackerley, R., Saar, K., McGlone, F., and Backlund Wasling, H. (2014). Quantifying the sensory and emotional perception of touch: differences between glabrous and hairy skin. Front Behav Neurosci *8*, 34.

S25. Loken, L.S., Evert, M., and Wessberg, J. (2011). Pleasantness of touch in human glabrous and hairy skin: order effects on affective ratings. Brain Res *1417*, 9-15.
